# Surface Co-Expression of Two Different PfEMP1 Antigens on Single *Plasmodium falciparum*-Infected Erythrocytes Facilitates Binding to ICAM1 and PECAM1

**DOI:** 10.1371/journal.ppat.1001083

**Published:** 2010-09-02

**Authors:** Louise Joergensen, Dominique C. Bengtsson, Anja Bengtsson, Elena Ronander, Sanne S. Berger, Louise Turner, Michael B. Dalgaard, Gerald K. K. Cham, Michala E. Victor, Thomas Lavstsen, Thor G. Theander, David E. Arnot, Anja T. R. Jensen

**Affiliations:** 1 Centre for Medical Parasitology, Department of International Health, Immunology & Microbiology, Faculty of Health Sciences, University of Copenhagen and Department of Infectious Diseases, Copenhagen University Hospital (Rigshospitalet), Copenhagen, Denmark; 2 Institute of Infection and Immunology Research, School of Biology, University of Edinburgh, Edinburgh, Scotland, United Kingdom; Case Western Reserve University, United States of America

## Abstract

The *Plasmodium falciparum* erythrocyte membrane protein 1 (PfEMP1) antigens play a major role in cytoadhesion of infected erythrocytes (IE), antigenic variation, and immunity to malaria. The current consensus on control of variant surface antigen expression is that only one PfEMP1 encoded by one *var* gene is expressed per cell at a time. We measured *var* mRNA transcript levels by real-time Q-PCR, analysed *var* gene transcripts by single-cell FISH and directly compared these with PfEMP1 antigen surface expression and cytoadhesion in three different antibody-selected *P. falciparum* 3D7 sub-lines using live confocal microscopy, flow cytometry and *in vitro* adhesion assays. We found that one selected parasite sub-line simultaneously expressed two different *var* genes as surface antigens, on single IE. Importantly, and of physiological relevance to adhesion and malaria pathogenesis, this parasite sub-line was found to bind both CD31/PECAM1 and CD54/ICAM1 and to adhere twice as efficiently to human endothelial cells, compared to infected cells having only one PfEMP1 variant on the surface. These new results on PfEMP1 antigen expression indicate that a re-evaluation of the molecular mechanisms involved in *P. falciparum* adhesion and of the accepted paradigm of absolutely mutually exclusive *var* gene transcription is required.

## Author Summary

*Plasmodium falciparum* is the most pathogenic human malaria parasite and its virulence has been linked to its capacity to express different adhesion proteins that enable the developing parasitized erythrocyte to bind to capillaries of the host, thereby avoiding removal by the spleen. Each parasite has approximately 60 genes encoding different versions of this adhesion protein, and a switch in surface display of these proteins enables the parasite to evade the immune system. Here we show that different variants of these binding proteins can be found expressed simultaneously on single infected red blood cells mediating binding to different endothelial receptors.

## Introduction

*Plasmodium falciparum* is the most pathogenic human malaria parasite and a major cause of morbidity and mortality in Africa. Its pathogenesis is closely associated with the adhesive properties of the variable erythrocyte surface antigens, PfEMP1, encoded by the *var* gene family [1]. These adhesins force erythrocytes infected with the parasite to bind to host receptors such as CD36, CD31/PECAM1 and CD54/ICAM1 on the endothelial lining of venular capillaries [2]–[5]. The more mature, replicating stages of the parasite thus leave the peripheral circulation, perhaps to avoid elimination by the reticulo-endothelial system of the host spleen. Their sequestration damages the host, as occlusion and inflammation around capillaries results in damage to vital organs and sometimes leads to potentially fatal complications such as cerebral malaria [1], [6], [7].

The highly polymorphic *var* genes are concentrated at the 28 chromosome telomeres, with a minority of loci in more central regions [8]. Switching expression of PfEMP1 antigens with alternative antigenic and cytoadherent properties allows *P. falciparum* to evade acquired immune responses and maintain infections despite antibody-mediated immune pressure [1], [9].

Based on distinct types of promoter sequences and their internal versus telomeric chromosomal location, the 60 member *var* gene family of the sequenced 3D7 clone can be classified into related groups [10], [11]. There are three major groups, (A, B, and C), two intermediate groups (B/A and B/C) and the more distantly related *var1* and *var2csa* genes. Mutation and recombination have generated a vast repertoire of polymorphic variants and how this antigenic variation system operates during infection is a major question in malaria biology and clinical research.

The interpretation of experiments testing the relationship between *var* gene transcription and the antigenic and adhesive properties of IE has been that a limited number of *var* mRNAs, possibly only one, is ultimately expressed as surface antigen on individual infected erythrocytes [12]–[17]. Such “mutually exclusive” *var* gene expression would limit exposure of antigens to the immune system and thus serve to extend infection periods. This seems advantageous to a vector-borne parasite whose transmission is governed by unpredictable environmental conditions and host availability. Northern blots, nuclear run-on assays and some single-cell RT-PCR experiments clearly demonstrated transcription of several *var* genes in ring stages, sometimes [16]–[18], but not always showing a reduction in the extent of polygenic *var* transcription as rings develop into trophozoites [19], [20]. This has been explained as the result of mRNA extraction from cell populations rather than individual cells and by indications that there is a trend, as intra-erythrocytic development progresses, for “loose” multi-*var* locus transcription to decrease, and a single mRNA type to become predominant, this dominant transcript becoming the sole translated and exported PfEMP1 [16], [17], [21]. Recent nuclear run-on data using A4 parasites, antibody-selected for expression of the A4varICAM1 PfEMP1, and also parasites preselected for adhesion to CSA or CD36 have somewhat reinforced the experimental support for the existence of predominant transcripts and mutually exclusive processes of transcriptional control [22], [23].

Strictly exclusive expression is most strongly supported by the demonstration that transfection of *P. falciparum* with plasmids transcribing *var* promoters is followed by a shut down of all endogenous *var* transcription [24]–[28]. These experiments elegantly demonstrate that there must be some form of epigenetic memory of transcriptional status. However, although transcriptional memory is likely to be fundamental to the control of the *P. falciparum* antigenic variation system, it is less clear how well transfection mimics *in vivo* regulation of *var* gene transcription in wild-type cultures. Nor is it clear how mutually exclusive expression is naturally established e.g in parasites which have not been expressing *var* genes such as those emerging into the bloodstream from the liver.

A monoclonal antibody (Bc6), detecting the antigen encoded by the *A4varICAM1 gene* on the surface of A4 infected erythrocytes, has been used to correlate this *var* gene expression with its adhesion phenotype in several studies [14], [15], [18], [29], [30]. However, efforts to link other *var* transcripts to the surface expression of particular PfEMP1 on single infected erythrocytes have been hampered by difficulties in generating a broad repertoire of specific, surface-reactive anti-PfEMP1 antibodies.

In this work, we have generated a number of such antisera after immunising with recombinant-produced PfEMP1 domains. We have combined these with *in vitro* antibody-selected *P. falciparum* lines [31] to identify simultaneous surface expression of PFD1235w and PF11_0008 PfEMP1 antigens on single infected erythrocytes.

Given the potential importance of dual PfEMP1 expression *in vivo,* we also assessed the adhesion to endothelial cells of IE with either one or two different PfEMP1 variants on the cell surface. Parasites with dual expression of both the PFD1235w and PF11_0008 antigens bound to both CD54/ICAM1 and CD31/PECAM1 and showed a markedly increased binding of IE to human umbilical vein endothelial cells (HUVEC). This binding is reminiscent of the multiple receptor interactions shown to be important in other vascular adhesion interactions [32], [33].

Interestingly, the severity of malaria disease has previously been associated with adhesion of IE to multiple receptors rather than one and clinical isolates of *P*. *falciparum* will bind several receptors simultaneously [34]–[36]. The explanation previously put forward to explain this has been that distinct domains of a single PfEMP1 molecule are interacting with multiple host receptors [37]. To our knowledge, this is the first study to demonstrate that multi-receptor binding can be mediated by different PfEMP1 antigens that are co-expressed on the same infected erythrocyte surface.

## Results

### Surface expression of PFD1235w and PF11_0008 on erythrocytes infected with selected 3D7 parasites

The expression of particular PfEMP1 surface antigens may switch during the infection and is proposed to be mutually exclusive in the sense that any single infected erythrocyte expresses only one variant antigen at a time, on the IE surface [14], [16], [17], [38]. We have shown that 3D7 parasites in culture, pre-selected using IgG from semi-immune children are transcribing several *var* genes. However, human IgG selected parasitized erythrocytes predominantly expressed one Group A PfEMP1 antigen (PFD1235w) on the IE surface [39]. Using new rat and rabbit antisera specific for the proteins encoded by two different *var* genes, annotated as PFD1235w and PF11_0008, we re-examined *var* gene and PfEMP1 surface antigen expression in three different antibody-selected sub-lines of 3D7.

The 3D7_ PFD1235w_ sub-line was selected from 3D7Dodowa1 [31], [39] using an antisera targeting the DBL4γ domain of PFD1235w. The 3D7_PFD1235w/PF11_0008_ and 3D7_PF11_0008_ sub-lines were selected from 3D7 using antisera targeting the CIDR1α domain of PFD1235w and the CIDR2β domain of PF11_0008, respectively. Each was grown in culture for only 3–5 cycles after the end of the selection period prior to the assay. We immuno-stained parasites for flow cytometry using antisera targeting DBL1α-CIDR1α, CIDR1α, DBL3β, DBL4γ, DBL5δ, DBL5δ-CIDR2β of PFD1235w, DBL4β and CIDR2β of PF11_0008, and DBL5ε and DBL5ε-DBL6ε of VAR2CSA and also examined individual live un-fixed single invaded IE using confocal microscopy.

**Figure 1:**
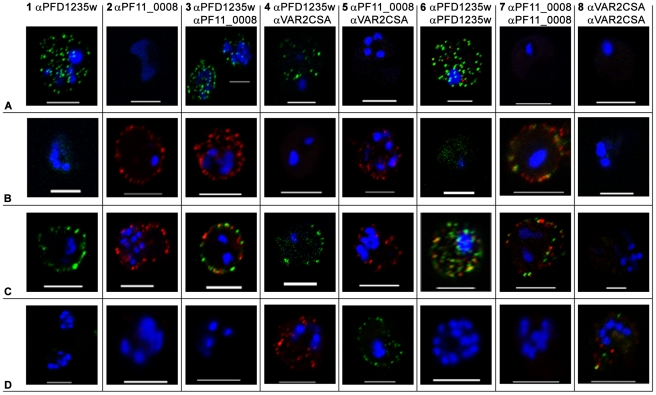
Surface expression of PfEMP1 on single 3D7 infected erythrocytes. (A) erythrocytes infected with a 3D7_PFD1235w_, (B) a 3D7_PF11_0008_, (C) a 3D7_PFD1235w/PF11_0008_, and (D) a NF54_ VAR2CSA_ sub-line. Localisation of PfEMP1 by confocal microscopy was done using (A1–D1) rat PFD1235w-DBL4γ antisera and (A2–D2) rabbit PF11_0008-CIDR2β antisera. Double surface staining was done using the following combinations (A3–D3) rat PFD1235w-DBL4γ and rabbit PF11_0008-CIDR2β antisera; (A4–D4) rat PFD1235w-DBL4γ and mouse VAR2CSA-DBL5ε antisera; (A5–D5) rabbit PF11_0008-CIDR2β and mouse VAR2CSA-DBL5ε antisera; (A6–D6) rat PFD1235w-DBL4γ and rabbit PFD1235w-CIDR1α; (A7–D7) rabbit PF11_0008-CIDR2β and rat PF11_0008-DBL4β; (A8–D8) rabbit VAR2CSA-DBL5ε-DBL6ε and mouse VAR2CSA-DBL5ε antisera. Antisera staining of PFD1235w expressed by the 3D7_PFD1235w_ (A1, A3, A4, A6) and 3D7_PFD1235w/PF11_0008_ (C1, C3, C4, C6) sub-lines was detected using a secondary antibody labelled with Alexa 488 (green). Antisera staining of PF11_0008 expressed by the 3D7_PF11_0008_ (B2, B3, B5, B7) and 3D7_PFD1235w/PF11_0008_ (C2, C3, C5, C7) was detected using a secondary antibody labelled with Alexa 568 (red). Staining of VAR2CSA expressed by NF54_VAR2CSA_ is red (D4, D8) and green (D5, D8). DAPI staining of DNA in the nuclei is blue. Scale bar 5 µm.

**Figure 2 ppat-1001083-g002:**
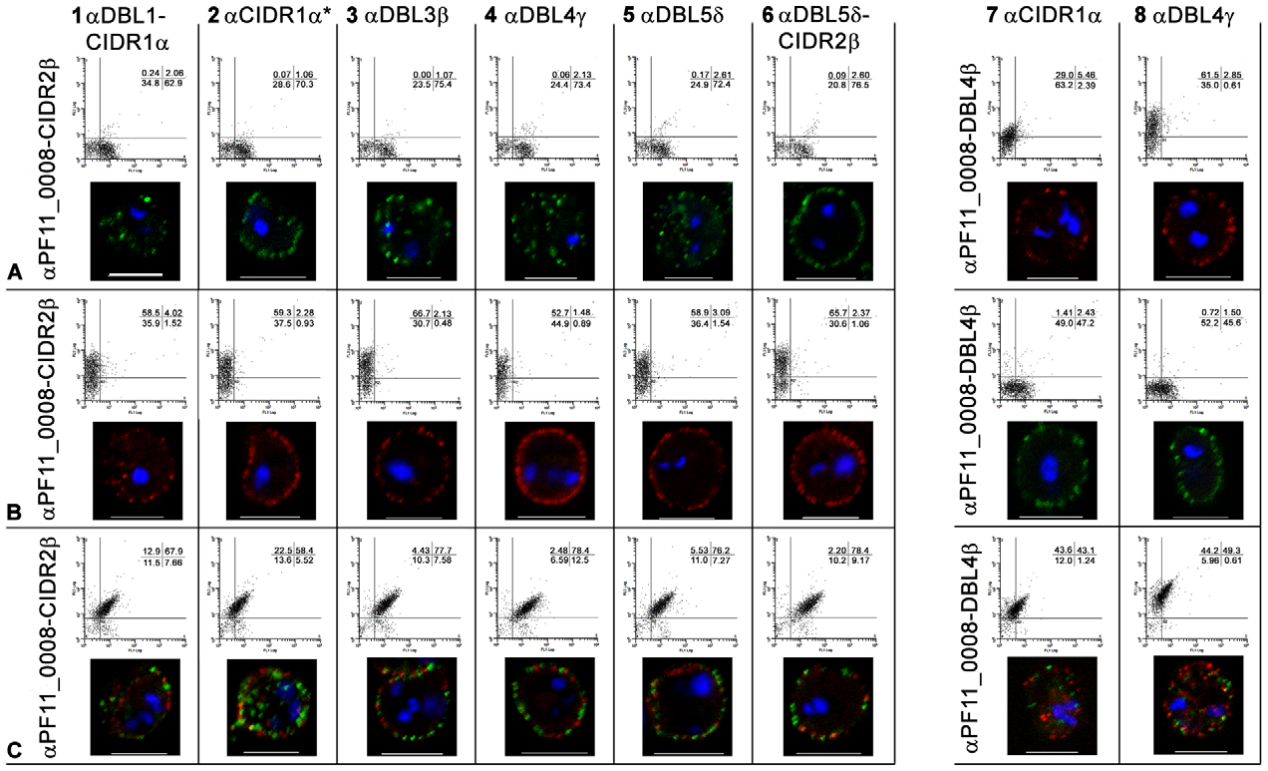
Simultaneous surface expression of PfEMP1 on single 3D7 infected erythrocytes. (A) erythrocytes infected with a 3D7_PFD1235w_, (B) a 3D7_PF11_0008_, and (C) a 3D7_PFD1235w/PF11_0008_ sub-line. Double surface staining and localisation of PfEMP1 by flow cytometry (dotplots) and confocal microscopy (photo inserts) was done using rabbit sera against CIDR2β of PF11_0008 in combinations with rat antisera against the following PFD1235w domains (A1–C1) DBL1α-CIDR1α, (A2–C2) CIDR1α*, (A3–C3) DBL3β, (A4–C4) DBL4γ, (A5–C5) DBL5δ, and (A6–C6) DBL5δ-CIDR2β. Similarly rat sera against DBL4β of PF11_0008 was used in combinations with rabbit sera against (A7–C7) CIDR1α and (A8–C8) DBL4γ of PFD1235w. For confocal microscopy rat antisera staining of PFD1235w expressed by the 3D7_PFD1235w_ (A1–A6) and 3D7_PFD1235w/PF11_0008_ (C1–C6) and PF11_0008 expressed by the 3D7_PF11_0008_ (B7–B8) and 3D7_PFD1235w/PF11_0008_ (C7–C8) sub-lines was detected using a secondary antibody labelled with Alexa 488 (green). Rabbit antisera staining of PF11_0008 expressed by the 3D7_PF11_0008_ (B1–B6) and 3D7_PFD1235w/PF11_0008_ (C1–C6) and PFD1235w expressed by the 3D7_PFD1235w_ (A7–A8) and 3D7_PFD1235w/PF11_0008_ (C7-C8) sub-lines was detected using a secondary antibody labelled with Alexa 568 (red). Flow cytometry plots include both infected and uninfected erythrocytes (see Materials and Methods). Flow cytometry settings and capture parameters for confocal microscopy were identical for all images. DAPI staining of DNA in the nuclei is blue. Scale bar 5 µm.

The majority of erythrocytes infected with the 3D7_PFD1235w_ sub-line stained positively in flow cytometry with all PFD1235w antisera (Figure S1A1–A8) without expression of any PF11_0008 (Figure S1A9 and S1A10). Live confocal microscopy of individual IE showed the characteristic punctate pattern of PfEMP1 antigen staining, indicating surface expression of the PFD1235w-encoded *var* gene (Figure 1A1, A3, A4, A6 and Figure 2A1–A8). No PF11_0008 (Figure 1A2, A3, A5, A7 and Figure 2A1–A8) or VAR2CSA (Figure 1A4, A5, A8) antigen staining was observed.

Similarly the majority of erythrocytes infected with the 3D7_PF11_0008_ sub-line stained positively with antisera targeting DBL4β and CIDR2β of PF11_0008 (Figure S1B9–10) without expression of PFD1235w (Figure S1B1–B8). Confocal microscopy agreed with the flow cytometric data, indicating expression of the PF11_0008 encoded *var* gene (Figure 1B2, B3, B5, B7 and Figure 2B1–8) without PFD1235w (Figure 1B1, B3–B4, B6 and Figure 2B1–8) or VAR2CSA (Figure 1B4, B5, and B8) antigen staining.

### The 3D7_PFD1235w/PF11_0008_ sub-line co-expresses PFD1235w and PF11_0008 on single infected erythrocytes

Contradicting the ‘mutually exclusive’ expression model [16], [17], [26], [27], single and single-infected erythrocytes infected by the 3D7_PFD1235w/PF11_0008_ sub-line of parasites stained positively with both of the differentially labelled antisera known to bind two different PfEMP1 antigens. In confocal microscopy the PFD1235w-DBL4γ and the CIDR2β PF11_0008 antisera showed an erythrocyte surface double staining pattern with punctate fluorescence, which showed limited or no co-localization (Figure 1C3 and Video S1).

The PFD1235w-DBL4γ, PFD1235-CIDR1α and PF11_0008- CIDR2β antisera used for selection do not cross-react with trypsin-resistant parasite surface antigens, as prior trypsinization of the IE abolished reactivity with all the antisera used in flow cytometry (Figure S2A–C). Blocking surface binding of these antisera with excess homologous antigen prior to staining of the sub-lines also indicated that neither antisera cross-reacted with other surface antigens on IE (Figure S2D–F).

To further exclude the possibility that the double staining of single IE was an artefact resulting from cross-reactivity with other surface antigens, we used various combinations of antisera with known specificity for different domains of the PfEMP1 encoded by the *PFD1235w* gene (DBL1α-CIDR1α, CIDR1α^*^, DBL3β, DBL5δ, DBL5δ-CIDR2β) and the *PF11_0008* gene (CIDR2β and DBL4β). All of the anti-PFD1235w and anti-PF11_0008 sera were specific for the 3D7_PFD1235w_ and the 3D7_PF11_0008_ sub-line, respectively (Figure S1). In combination with αPF11_0008-CIDR2β, each anti-PFD1235w antiserum also double stained the 3D7_PFD1235w/PF11_0008_ sub-line (Figure 2C1–C6). Similar data was obtained using αPF11_0008-DBL4β in combination with αPFD1235w-CIDR1α and DBL4γ (Figure 2C7–C8). These results also indicate that single erythrocytes infected by antibody-selected 3D7 are expressing more than one PfEMP1 antigen on individual erythrocyte membranes.

To further eliminate the possibility that ‘double antigen staining’ on single IE is an artefact related to the use of two different antisera, we tested a variety of additional combinations of antisera, each with different specificities. Combinations of antisera raised against PFD1235w-DBL4γ domain and antisera raised against the VAR2CSA-DBL5ε domain (Figure 1A4–D4) or anti-PF11_0008-CIDR2β sera with VAR2CSA-DBL5ε antisera were tested (Figure 1A5–D5). These combinations always showed a single staining phenotype when reacting with the 3D7_PFD1235w/PF11_0008_ IE and the other sub-lines of 3D7. Surface staining of the 3D7_PFD1235w_ sub-line using two differently fluorescently labelled antibodies, a rat antiserum raised against the DBL4γ domain and rabbit antiserum raised against CIDR1β domain of the same PFD1235w antigen also showed dual staining, with limited co-localisation and distinctly labelled spots of erythrocyte surface fluorescence (Figure 1A6 and C6). Similarly, staining of the 3D7_PF11_0008_ sub-line using differentially labelled antibodies targeting PF11_0008 DBL4β (rat) and CIDR2β (rabbit) (Figure 1B7 and C7) and staining of the 3D7_VAR2CSA_ sub-line using antibodies targeting DBL5ε (mouse) and DBL5ε-DBL6ε (rabbit) of VAR2CSA also showed the dual staining, without significant co-localization phenotype (Figure 1D8).

### Simultaneous surface expression of PFD1235w and PF11_0008 in clones of 3D7_PFD1235w/PF11_0008_ single infected erythrocytes

**Figure 3:**
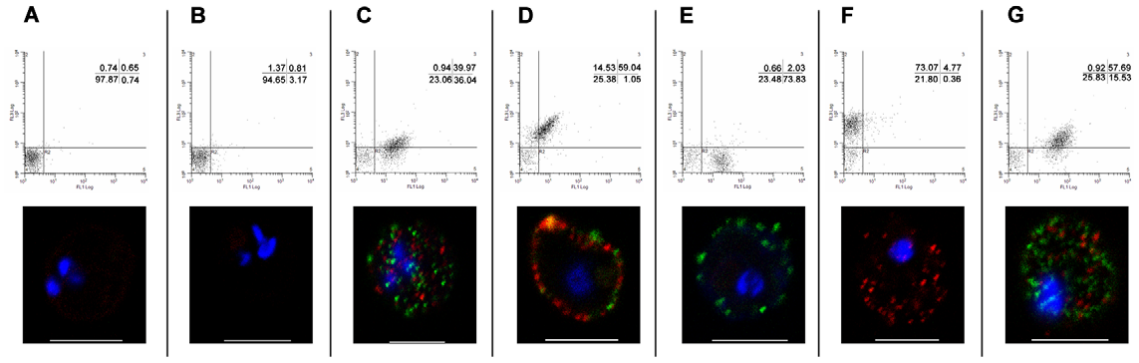
Simultaneous surface expression of PfEMP1 on 3D7_PFD1235w/PF11_0008_ clone 3 infected erythrocytes. Double surface staining and localisation of PfEMP1 by flow cytometry (dotplots) and confocal microscopy (photo inserts) was done using (A) buffer, (B) rat and rabbit pre-bleeds, (C) rat sera against PFD1235w-DBL4γ and rabbit sera against PF11_0008-CIDR2β, (D) rabbit sera against PFD1235w-DBL4γ and rat sera against PF11_0008-DBL4β, (E) rat sera against PFD1235w-DBL4γ and rabbit pre-bleed, (F) rat pre-bleed and rabbit sera against PF11_0008-CIDR2β, and (G) rat sera against PFD1235w-DBL5δ-CIDR2β and rabbit sera against PF11_0008-CIDR2β. For confocal microscopy rat antisera staining of PFD1235w (C, E, G) and of PF11_0008 expressed by the 3D7_PFD1235w/PF11_0008_ clone 3 (D) was detected using a secondary antibody labelled with Alexa 488 (green). Similarly rabbit antisera staining of PFD1235w (D) and PF11_0008 (C, F, G) was detected using a secondary antibody labelled with Alexa 568 (red). Flow cytometry plots include both infected and uninfected erythrocytes (see Materials and Methods).Flow cytometry settings and capture parameters for confocal microscopy were identical for all images. DAPI staining of DNA in the nuclei is blue. Scale bar 5 µm.

To obtain clonal lines of 3D7_PFD1235w/PF11_0008_ IE we carried out a limiting dilution cloning exercise, following which the resulting clones were grown for 7–10 cycles, prior to flow cytometry and confocal microscopy PfEMP1 surface expression analysis. Seven of nine clones originating from this experiment showed simultaneous surface expression of both PFD1235w and PF11_0008 as shown for clone 3 in Figure 3. The double expressing phenotype was only shown by a minority of the populations in the two remaining clones 4 and 8, indicating that the dual expression phenotype can be spontaneously lost over time as shown for clone 4 in Figure S3.

### Luminex, ELISA, flow cytometry and confocal microscopy testing of reagents used for the surface staining of IE

**Figure 4:**
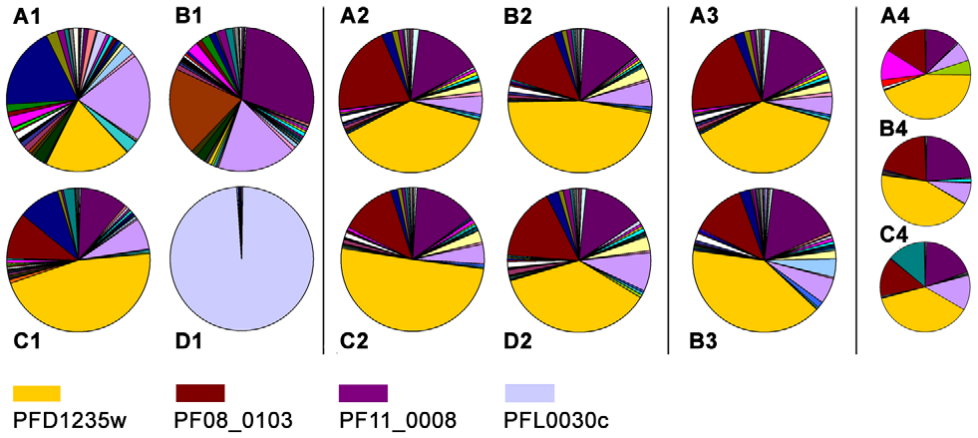
Individual *var* gene transcripts relative to the total *var* transcript copy number in ring-stage IE. The analysis was done on cultures of the (A1) 3D7_PFD1235w_, (B1) 3D7_PF11_0008_, (C1) 3D7_PFD1235w/PF11_0008_, and (D1) 3D7_VAR2CSA_ sub-lines. A similar analysis was done on Dynabeads antibody enriched 3D7_PFD1235w/PF11_0008_ (A2) IE prior to enrichment, (B2) IE enriched using rabbit PFD1235w-DBL4γ antisera, (C2) IE enriched using rabbit PFD1235w-CIDR1α antisera. (D2) IE enriched using rabbit PF11_0008-CIDR2β antisera. In another experiment we used unsorted (A3) 3D7_PFD1235w/PF11_0008_ IE and (B3) FACS sorted 3D7_PFD1235w/PF11_0008_ IE stained using rabbit PF11_0008-CIDR2β and rat PFD1235w-DBL4γ antisera followed by FITC- and Alexa Fluor 610-R-PE conjugated secondary antibodies as described in Materials and Methods. The 3D7_PFD1235w/PF11_0008_ sub-line was cloned by limiting dilution and the transcript profile of nine different clones was analysed. The transcript profiles of three representative clones are shown (A4-C4).

Affinity purified antisera raised in rats against PFD1235w-DBL4γ and in rabbits against CIDR2β of PF11_0008, as well as rabbit antisera against PFD1235w-CIDR1α, were depleted of any anti-V5-HIS fusion protein reactivity. The specificity of the resulting antibodies was then assessed by both ELISA and a bead-based Luminex assay [40]. We found the three depleted antibody preparations to be specific for the homologous immunizing protein (Figure S4) as none cross-reacted with any of the 48 heterologous PfEMP1 domains in the Luminex (Figure S4D–F). The Luminex assay and ELISA both included the CIDR1α domain of PF08_0103, i.e. representing a *var* gene transcript known to be highly abundant in the 3D7_PFD1235w/PF11_0008_ IE tested (Figure 4).

Having tested the specificity of the antisera and of the affinity purified and depleted antibodies, we repeated the surface staining experiments presented in Figure 1 and Figure S1 and the double staining experiments shown in Figure 1 and Figure 2, on aliquots of the same batch of parasites, with the affinity purified and tag-depleted antibodies (Figure S5). The tag-depleted PFD1235w antibodies surface stained the 3D7_PFD1235w_ and the 3D7_PFD1235w/PF11_0008_ sub-lines, but not the 3D7_PF11_0008_ line. Similarly, the depleted PF11_0008 antibodies surface stained the 3D7_PF11_0008_ and the 3D7_PFD1235w/PF11_0008_ sub-lines, but not the 3D7_PFD1235w_ line. All antibody purifications showing identical punctate PfEMP1 surface staining patterns to those observed with the complete antisera used in Figure 1 and Figure 2. Likewise, flow cytometry data using purified and depleted antibodies gave the same results as obtained using crude antisera (Figure S1 and Figure S5).

### Transcription of *var* genes in the 3D7 sub-lines

In parallel to the antibody-based PfEMP1 surface antigen detection studies, we measured *var* gene transcript levels using primer sets targeting each of the 58 active *var* genes and two pseudo *var* genes of the 3D7 genome [41]–[43]. Only the NF54_VAR2CSA_ sub-line culture (Figure 4D1), showed exclusive transcription of a single *var* gene (*var2csa*). All other sub-lines showed the presence of several major transcripts during the ring-stages, the most transcriptionally active stages for *var* gene expression (Figure 4A1–C1). The 3D7_PFD1235w_ sub-line culture transcribed three different *var* genes in similar quantities; *PFD1235w* (Group A), *MAL6P1.316* (Group A/B), and *PFD0625c* (Group C). The three most abundant *var* transcripts in the 3D7_PFD11_0008_ sub-line cultures were *PF11_0008* (Group A), *PF07_0050* (Group B/C), and *PFD0625c* (Group C). In the 3D7_PFD1235w/PF11_0008_ sub-line cultures the three most abundant transcripts were *PFD1235w* (Group A), *PF11_0008* (Group A), and *PF08_0103* (Group B/C).

*PFD1235w* and *PF11_0008* constituted 19% and 1%, respectively, of the total *var* transcripts in the 3D7_PFD1235w_ sub-line (Figure 4A1). They constituted 1% and 29%, respectively, in the 3D7_PF11_0008_ sub-line (Figure 4B1) and 46% and 11%, respectively, in the 3D7_PFD1235w/PFD11_0008_ sub-line (Figure 4C1).

To verify that the 3D7_PFD1235w/PFD11_0008_ culture is a homogenous population we did different experiments to enrich for PFD1235w and PF11_0008 double positive IE. Populations of 3D7_PFD1235w/PFD11_0008_ were enriched using antisera (αPFD1235w-DBL4γ, αPFD1235w-CIDR1α, and αPF11_0008-CIDR2β) bound to Protein A-coupled Dynabeads, FACS sorted using αPFD1235w-DBL4γ and αPF11_0008-CIDR2β simultanously in addition to cloning by limiting dilution as described above.

The Dynabeads-enriched and FACS sorted IE were grown for less than one cycle and RNA was extracted from the ring stage. Extracted mRNA from these stages showed the presence of several major *var* transcripts (Figure 4B2–D2 and Figure 4B3), a profile similar to that seen in mRNA extracted from ring-stages of the unsorted 3D7_PFD1235w/PFD11_0008_ IE in Figure 4C1, 4A2, and 4A3.

Three different representative 3D7_PFD1235w/PFD11_0008_ clones (1, 3, and 5) originating from the limiting dilution experiment showed an almost identical transcript profile with the presence of several major transcripts (Figure 4A4–C4). This indicates the unsorted 3D7_PFD1235w/PFD11_0008_ culture largely is a homogeneous population, a conclusion also supported by the flow cytometry data (Figure 2C1–C8).

### Full length transcription of *PFD1235w and PF11_0008*

**Figure 5:**
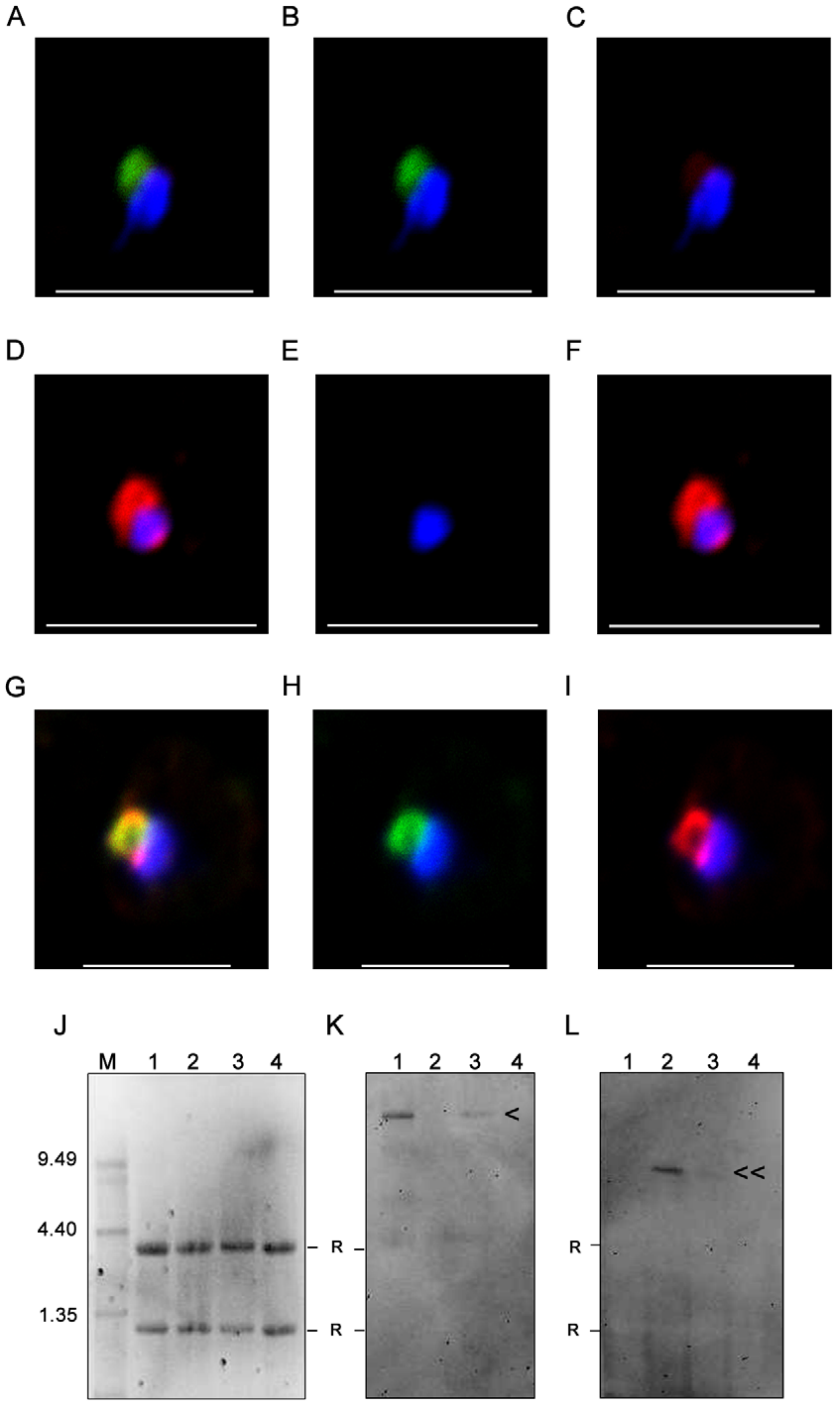
Nuclear transcripts of *PFD1235w* and *PF11_0008* in single erythrocytes. FISH analysis was done on single ring stage parasites in 100 single-cells of (A–C) 3D7_PFD1235w_ IE, (D–F) 3D7_PF11_0008_ IE and (G–I) 3D7_PFD1235w/PF11_0008_ clone 3 IE using a (B, E, H) *PFD1235w* (green) and (C, F, I) *PF11_0008* specific probe (red). The overlaying of the two colours identified transcripts of *PFD1235w* in (A) 3D7_PFD1235w_ IE, *PF11_0008* in (D) 3D7_PF11_0008_ IE, and simultaneous transcription of *PFD1235w* and *PF11_0008* in (G) 3D7_PFD1235w/PF11_0008_ clone 3 IE. Ethidium bromide staining (J) and Northern blotting (K and L) verification of the specificity of the probes used for FISH was done on RNA purified from 3D7 sub-lines transcribing *PFD1235w* (J1, K1, L1), *PF11_0008* (J2, K2, L2), co-transcribing *PFD1235w* and *PF11_0008* (J3, K3, L3), and FCR3 transcribing *var2CSA* (J4, K4, L4). The blots were probed with (K) a *PFD1235w* and (L) a *PF11_0008* specific probe. M: RNA molecular weight marker; < *PFD1235w* transcript (10.66 kb); ≪ *PF11_0008* 8.98 kb transcript. R: ribosomal band. Scale bar 5 µm.

Full length transcription of *PFD1235w* and *PF11_0008* and all the *var* genes listed in Table S2 was tested using specific cross-intron primers. In real-time quantitative PCR assays with cDNA synthesised from total RNA extracted from the three 3D7 sub-lines and our clones we obtained similar Ct-values, whether using primers targeting exon I, or primers spanning the intron regions (Figure S6). This, in addition to Northern blotting (Figure 5K and 5L), indicates that these genes are being transcribed into full length mRNA species by the parasites. In addition, sequencing of the introns of both *PFD1235w* and of *PF11_0008* (using genomic DNA from selected sub-line and clonal IE as template) showed they were intact and identical to the genomic sequences available at http://plasmodb.org/plasmo/. Sequencing of cDNA also showed the introns of *PFD1235w, PF11_0008,* and the introns of the *var* genes in Table S2, were correctly spliced out of the mature mRNA.

### FISH and Northern blotting analysis of *PFD1235w and PF11_0008* transcripts

Single-cell *PFD1235w* and *PF11_0008* transcription of the two *var* genes located on chromosome 4 and 11 was further analysed by using RNA-FISH *in situ* hybridization to *var* gene mRNA using appropriate probes. The FISH indicates that 65–80% of single ring-stage nuclei of parasites taken from cultures of the 3D7_PFD1235w_ and 3D7_PF11_0008_ sub-lines transcribe either *PFD1235w* (Figure 5A–C) or *PF11_0008* (Figure 5D–F). None of the 3D7_PFD1235w_ IE showed staining with the PF11_0008 probe and vice versa.

In agreement with the antibody-mediated PfEMP1 antigen surface detection data (Figures 1–3, Figure S1, Figure S3 and Figure S5) and the real-time quantitative PCR data (Figure 4 and Figure S6) *PFD1235w* and *PF11_0008* could both be detected as hybridising mRNA species being transcribed in 99 of 100 different single nucleated cells of the 3D7_PFD1235w/PF11_0008_ clone 3 (Figure 5G–I). The specificity of the probes for the two differently sized PfEMP1 mRNA species was further confirmed by Northern blotting (Figure 5K and 5L) and the identified length of mRNA agrees with that predicted as the transcript length from their respective sequenced genes (http://plasmodb.org/plasmo/). Control slides pre-treated with RNAse prior to hybridization were all negative.

### When co-expressed on single IE, the PFD1253w and PF11_0008 PfEMP1 antigens mediate binding to two different endothelial receptors

**Figure 6:**
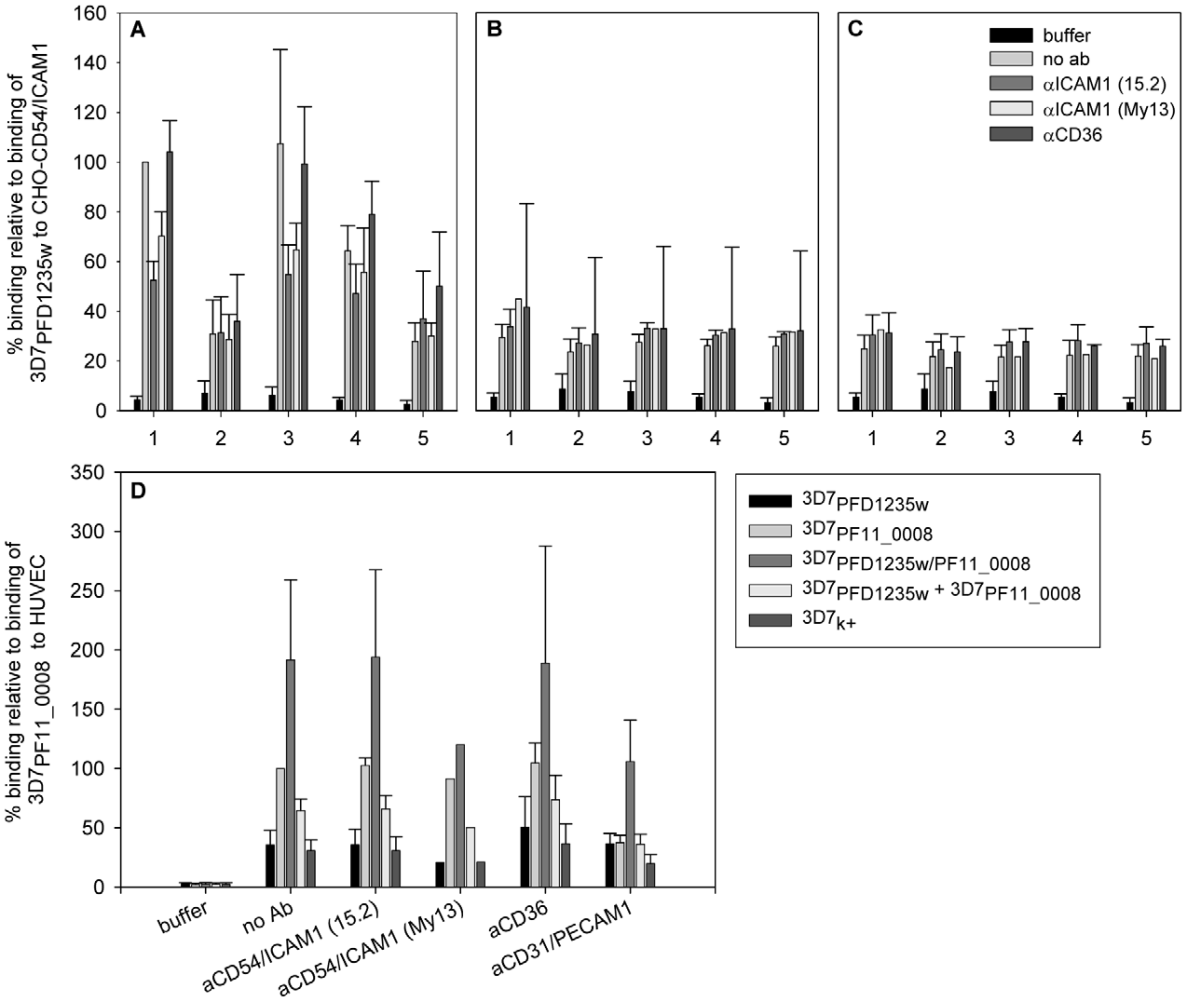
Infected erythrocytes bind CD54/ICAM1, CD31/PECAM1 or both receptors. Binding of (1) 3D7_PFD1235w_, (2) 3D7_PF11_0008_, (3) 3D7_PFD1235w/PF11_0008_, (4) 3D7_PFD1235w_ mixed 1∶1 with the 3D7_PF11_0008_ sub-line and (5) non selected knob positive 3D7 (3D7k+) to (A) CHO expressing CD54/ICAM1, (B) CHO expressing CD36, or (C) wildtype CHO cells was done in the absence or presence of anti-CD54/ICAM1 (clone 15.2 and My13) and anti-CD36 antibodies. Similarly binding of the different 3D7 sub-lines to (D) human umbilical vein endothelial cells (HUVEC) was done in the absence or presence of anti-CD54/ICAM1 (clone 15.2 and My13), anti-CD36 or anti-CD31/PECAM1 antibodies. The data represents the mean of at least three independent experiments except for the αICAM1 (My13) experiment in B, C, and D which was only done once. Error bars are ± SD. Buffer: background control with no IE added; no Ab: binding assay done using IE, but without addition of antibodies.

Having established simultaneous expression of two species of PfEMP1 on the IE surface, we then analysed whether this dual expression affects the binding phenotype of the infected erythrocytes (Figure 6). 3D7_PFD1235w_ infected erythrocytes showed specific binding to CD54/ICAM1 transfected CHO cells (Figure 6A) and no binding to CHO-CD36 cells (Figure 6B) or wild type CHO cells (Figure 6C). The observed binding was specifically inhibited by anti-CD54/ICAM1 (15.2 and My13) antibodies (Figure 6A) which have been shown to inhibit binding of PfEMP1 to ICAM1/CD54 [44].

Erythrocytes infected with 3D7_PF11_0008_ did not bind CD54/ICAM1 (Figure 6A). The 3D7_PFD1235w/PF11_0008_ sub-line, which expressed both PFD1235w and PF11_0008, bound as strongly to CD54/ICAM1 as the PFD1235w infected cells which express only this ICAM1–binding antigen (Figure 6A). The 3D7_PF11_0008_ sub-line bound specifically to the un-stimulated HUVEC cells which constitutively express PECAM1/CD31 and von Willebrand factor. This binding was markedly reduced in the presence of antibodies to CD31/PECAM1 (Figure 6D), indicating that CD31/PECAM1 was important for PfEMP1-mediated binding in this assay. Cells expressing PFD1235w also showed very slight binding to un-stimulated HUVEC cells, probably explicable by a low level expression of CD54/ICAM1 on these cells as this binding was slightly reduced by CD54/ICAM1 antibodies (Figure 6D).

However cells expressing both PFD1235w and PF11_0008 bound strongly to the HUVEC cells, with binding levels 2–3 times higher than the binding of the cells expressing PF11_0008 only. This binding could only be partially inhibited by anti-CD31/PECAM1 and CD54/ICAM1 antibodies. This result indicates that cells co-expressing PFD1235w and PF11_0008 can bind both CD54/ICAM1 and CD31/PECAM1, whereas IE having only one of the two PfEMP1 species on the surface bind to one or another of the two receptors, but not both. Interestingly the binding of the 3D7_PFD1235w/PF11_0008_ sub-line to HUVEC cells (Figure 6D) was stronger than the binding of the 3D7_PF11_0008_ sub-line. This could reflect an additive effect of PfEMP1 co-expression.

## Discussion

The complete *P. falciparum* 3D7 genome sequence and real-time quantitative RT-PCR enable accurate measurement of the relative amounts of *var* gene transcripts in intra-erythrocytic malaria parasite populations. To further analyse the relationship between *var* gene transcripts and the individual infected erythrocyte's PfEMP1 antigen surface expression and cytoadhesion phenotype, we have combined mRNA quantification and FISH analysis with FACS and single-cell live confocal microscopy using PfEMP1 antisera of demonstrated specificity in addition to *in vitro* adhesion assays, in antibody-selected, but otherwise genetically unmodified parasites. The primary intention was to test the ‘mutually exclusive expression’ hypothesis for the *P. falciparum* PfEMP1 mediated antigenic variation system.

A handful of studies have investigated this relationship between *var* transcripts and its eventual outcome in terms of surface PfEMP1 expression and adhesion. These have used parasites selected for rosetting [16], [45], adhesion to ICAM1 or CSA [15], [17], [19] or selected using the *A4varICAM1* monoclonal antibody Bc6 [15], [46]. Data supporting exclusive antigen expression on IE has been obtained using ‘pan-reactive’ antibodies targeting the conserved Acidic Terminal Sequence of PfEMP1, which detected single PfEMP1-sized bands in immuno-precipitations and Western blots [16]. However, similar antibodies have also shown protein expression of several differently sized PfEMP1 bands in other experiments with adhesion-selected clonal populations [19] and immune sera-selected cultures are also known to express several species of PfEMP1 [39]. Additionally, a more recent study using a transfected A4 parasite line show surface co-expression of miniPfEMP1 protein with endogenous PfEMP1 [47].

Direct demonstration of PfEMP1 using gel electrophoretic methods is technically difficult due to the low amounts of PfEMP1 on parasitized erythrocytes and the fact that Western blot-based typing is rarely possible because typing sera usually recognise conformational epitopes and not denatured blotted PfEMP1. However, flow cytometry and confocal microscopy can detect native PfEMP1 conformations on live cells, at both cell and population level. We used this to analyse PfEMP1 surface expression on antibody-selected IE populations known to predominantly, but not exclusively, transcribe the *PFD1235w* and *PF11_0008 var* genes.

The ring-stages of antibody selected 3D7-derived lines showed polygenic *var* transcription with several abundant *var* mRNAs (Figure 4A1–C1, Figure 4A2, and Figure 4A3). Dynabeads sorted (Figure 4B2–D2), and FACS sorted lines (Figure 4B3) also showed polygenic *var* gene transcription. The real-time Q-PCR data are also supported by RNA-FISH and Northern blot analysis showing co-transcription of full-length *PFD1235w* and *PF11_0008 var* genes associated with single-cell nuclei of 3D7_PFD1235w/PF11_0008_ (Figure 5).

By contrast, the NF54_VAR2CSA_ sub-line had a single dominant gene, *var2csa,* whose transcript was 98% of total *var* mRNA (Figure 4D1). The dominant, exclusive *var2csa* transcription of repeatedly CSA selected parasites [41], [42], [48] is the best demonstration of ‘mutually exclusive’ *var* transcription. However, it is also to some extent a special case. The *var2csa* gene has its own promoter, *upsE* [10], and an unique upstream open reading frame involved in the control of *var2csa* expression [49] and thus its regulation has certain non-standard features [42]. Strictly monoallelic or unilocus expression of the *var2csa* gene has however, also been questioned by recent publications showing transcription of duplicated *var2csa* genes [50], which appears to be simultaneous in individual parasites [51]. However dual expression of both VAR2CSA variants on the erythrocyte surface has not been shown.

We observe neither monoallelic mRNA transcription, nor monomorphic antigen expression at the erythrocyte surface in individual 3D7_PFD1235w/PF11_0008_ IE. These cells clearly reacted with more than one specific anti-PfEMP1 antisera. Other selected sub-lines reacted exclusively with either antisera (Figure 1, Figure 2, Figure S1 and Figure S5). In our assays these antisera were highly specific for the immunizing antigen (Figure S1, S2, and S4). Our experiments appear to exclude the possibility that these antisera are non-specifically reacting with other surface expressed parasite antigens. We consider that these novel results indicate that, contrary to the current consensus, at least two different PfEMP1 antigens can in some circumstances be expressed on the surface of a single 3D7 IE.

A puzzling phenomenon associated with PfEMP1 surface staining with antigen and domain specific antisera remains. This is the observation that differentially labelled antisera raised against separate domains (Figure 1) or identical domains (unpublished data) of the same PfEMP1 protein consistently show non-colocalizing patterns of punctate surface fluorescence. This could be the result of some PfEMP1 topological phenomena, or more likely an artificial outcome of the experimental setup with cross-linking of primary via secondary species-specific antibodies [52].

Disregarding our flow cytometry and single-cell microscopy data the presence of several abundant full-length *var* mRNAs in the three antibody selected sub-lines (Figure 4A1–C1, Figure 4A2, Figure 4A3) could be explained by that fact that mRNA was extracted from *in vitro* cultured cell populations rather than individual cells. To exclude this possibility we did limiting dilution of the 3D7_PFD1235w/PF11_0008_ sub-line to obtain a clonal population of cells. From this experiment nine clones were obtained of which seven double positive clones show a slightly reduced complexity, but still multiplicity of *var* gene transcripts (compare Figure 4A4–C4 and Figure 4C1). Thus, the multiple *var* transcripts seen (Figure 4C1, Figure 4A2, and Figure 4A3) can not be explained by our use of a heterogeneous cell population.

At the genetic level, several DNA sequences required for appropriate *var* gene control have been reported e.g. the pairing of a 5′*var* promoter with an intronic promoter at the 3′end of the same *var* gene [24]. We therefore screened for potential confounding defects in our parasite lines and in clone 3. Using cross-intron primers for the abundantly transcribed *var* genes we found correct splicing of exon I to exon II. In addition, the real time Q-PCR for each abundant *var* gene transcript indicated similar transcript levels of several full-length *var* mRNAs in the early stage IE (Figure S6). Sequencing the intronic regions of the *PFD1235w* and *PF11_0008 var* genes located on two different chromosomes revealed that these were identical to the sequences available on PlasmoDB. Thus, constitutive transcription by single 3D7_PFD1235w/PF11_0008_ IE due to some defect in their respective intron sequences does not explain our dual surface expression data. In addition as shown by flow cytometry, two of the clones switched surface expression during culturing with a minor population still co-expressing PFD1235w and PF11_0008 and a majority expressing unidentified PfEMP1s (Figure S3). This strongly indicates a shut-down of PFD1235w and PF11_0008 PfEMP1 surface expression and further indicates constitutive transcription does not explain our dual surface staining data.

Whether the simultaneous expression of more than one PfEMP1 occurs in single IE during natural infections is not known as we currently have a better understanding of malaria antigen variation *in vitro* than *in vivo*. In natural infections, *P. falciparum* sequesters in deep tissue prior to the onset of cell division and under physiological blood flow sequestration is mediated by avidity-dependent binding to multiple host-receptors, mimicking the process of leukocyte recruitment [53]–[55]. Antibodies to CD36 have been shown to reduce rolling and adhesion of IE, residual rolling being further inhibited by antibody to ICAM1 [55]. These receptors operate synergistically to mediate strong cytoadherence when coexpressed on endothelial cells [56].

PfEMP1 proteins have multiple domains [8] and most CIDR-α type domains bind CD36 [57], while some DBLβc2 domains bind ICAM1 [58]–[61] and some single PfEMP1 species have been shown to mediate multiple independent interactions with a diverse set of host receptors including CD31/PECAM-1, the blood group A antigen, normal nonimmune IgM, heparan sulfate–like glucosaminoglycan, and CD36 [62].

A study has suggested that the ability of parasites to bind to multiple receptors is correlated with disease severity [63]. In addition, several lines of evidence have implicated CD54/ICAM1 [64]–[67], CD31/PECAM1 [68] as well as PFD1235w and PF11_0008 [39], [69], [70] as having a role in severe disease. Interestingly, we found the PFD1235w and PF11_0008 surface co-expressed on the 3D7_PFD1235w/PF11_0008_ sub-line to mediate binding to CD54/ICAM1 and CD31/PECAM1, respectively (Figure 6). To our knowledge this is the first study to demonstrate dual receptor binding in malaria cytoadherence being mediated by two different PfEMP1 molecules on the surface of single IE. A potential mechanism for the two PfEMP1 interactions could involve a role for PFD1235w as a primary ligand for rolling and CD54/ICAM1 binding on the endothelium thus enabling further contacts with CD31/PECAM1 which could be mediated by PF11_0008, when both are present on the IE surface. Adhesion would then be improved through simultaneous binding to several receptors as indicated by our data in Figure 6. This process may be similar to that of lymphocyte rolling based on carbohydrate-receptor interactions [71].

If more than one PfEMP1 antigen is expressed on individual IE in *in vivo* infections it may be most advantageous during the immediately post-hepatocytic establishment phase. The first post-hepatocytic generation lacks epigenetic memory of *var* gene transcription and translation. Relaxed transcription [72] and translation may thus be initially unavoidable. It may also ensure the highest avidity binding interaction possible and rapid sequestration. Transcription of those *var* genes expressed by successfully sequestering survivors of the post-hepatocytic wave of infection would epigenetically mark these *var* genes for expression in their descendent population, whilst leaving their unexpressed and thus unmarked *var* genes to become silenced. Such an ‘early loose-tight late’ model for *var* gene transcription in blood stage infection is compatible with experiments demonstrating that transcription of a particular *var* gene promoter leads to the silencing of the other *var* genes in the repertoire [73].

Our *var* gene transcript level, PfEMP1 surface expression, and cytoadhesion data may be indicating that the primary role of the *P. falciparum var* genes is the sequestration reaction and that at least in the earliest phases of blood stage infection, escape of IE from the circulation to a sequestration site which ensures successful replication takes precedence over shielding the antigenic variation repertoire from immune surveillance. The tiny amounts of PfEMP1 present at very low parasitaemia are unlikely to be sufficient to promote protective seroconversion. As infections develop and variant-specific parasitaemia rises, the repertoire become protected by epigenetic silencing until finally a novel cytoadhesion phenotype expressing a new PfEMP1 variant outgrows the increasingly immunocompromised founder population and the process starts over.

In summary, we have shown protein expression of two different *var* genes on the membrane of single erythrocytes infected with *P. falciparum* 3D7 to facilitate cytoadhesion of the infected cells to two different human receptors. These observations contradicts the hypothesis of mutually exclusive PfEMP1 expression and at the same time offers an additional molecular explanation for how individual IE can mediate adhesion to multiple host receptors.

## Materials and Methods

### Malaria parasites and *in vitro* selection procedure

The *P. falciparum* isolate NF54 and the NF54 derived clone 3D7 were cultured in blood group 0 erythrocytes as previously described [74]. Cultures were routinely genotyped by PCR using primers targeting the polymorphic loci MSP2 and Glurp as described [75] and mycoplasma tested using the MycoAlert Mycoplasma Detection Kit (Lonza) following the manufactures instructions.

IgG from rabbits immunized with DBL4γ or CIDR1α of 3D7 *PFD1235w* and CIDR2β of 3D7 *PF11_0008* were used to obtain three different sub-lines of 3D7 parasites. 3D7_PFD1235w,_ originating from a previously selected line referred to as 3D7Dodowa1 [31] was obtained using sera targeting a PFD1235w-DBL4γ recombinant antigen. 3D7_PF11_0008_ originating from 3D7 was selected using sera targeting the CIDR2β of *PF11_0008* and 3D7_PFD1235w/PF11_0008_ similarly originating from 3D7 was obtained using sera targeting a CIDR1α domain recombinant protein of *PFD1235w*. NF54_VAR2CSA_ kindly provided by Morten A. Nielsen was selected using rabbit antisera raised against a DBL5ε-6ε recombinant protein based on the *var2csa* gene. Briefly, the different rabbit sera were depleted on human uninfected erythrocytes type 0, incubated with gelatine purified trophozoite-stage 3D7/NF54 parasites for 30 min at 37°C, and unbound antibodies were removed by washing. Subsequently, IE were incubated with Protein A-coupled Dynabeads (Invitrogen) for 30 min at 37°C and bound IE were trapped using a magnet. Trapped IE were transferred to new culture flasks for continued *in vitro* culturing and the procedure was repeated until cultures stained positive by the selecting antisera in flow cytometry.

### Dynabeads sorting of parasites

Gelatine purified 3D7_PFD1235w/PF11_0008_ IE was incubated for 30 min at 37°C with rabbit sera targeting (PFD1235w-DBL4γ, PFD1235w-CIDR1α, and PF11_0008-CIDR2β) and incubated with Protein A-coupled Dynabeads. Bound IE were trapped as described above, grown for less than one cycle to rings, following which RNA was purified, reverse transcribed and used for real-time quantitative PCR.

### Flow cytometry sorting of parasites

MACS purified 3D7_PFD1235w/PF11_0008_ IEs were double stained with rat PFD1235w-DBL4γ and rabbit PF11_0008-CIDR2β antisera as described below and analysed on a FACSAria (Becton Dickinson). A total of 1×10^6^ double positive IE were collected and grown for less than one cycle to rings, following which RNA was purified, reverse transcribed and used for real-time quantitative PCR.

### Limiting dilution of parasites

Late stage 3D7_PFD1235w/PF11_0008_ IE was obtained by MACS purification and cloned using a slightly modified version of the protocol described by Walliker and Beale [76]. In brief, 0.25 or 0.5 IE in a total volume of 100 µl RPMI 1640 (Lonza) with 1% hematocrit, 5 mg/ml Albumax II (Life Technologies, 0.18 mg/ml glutamine (Sigma-Aldrich), 0.05 mg/ml gentamicin (Gibco) and 10% Normal Human Serum were seeded into each well of flat bottomed 96-wells plates (NUNC). New media was added every second day and additional 1% hematocrit was added on day 5. A total of 64 wells were checked for the presence of IE by Giemsa staining on day 12 and16 yielding nine different clones. RNA was purified from all nine clones for real-time quantitative PCR analysis and the PFD1235w and PF11_0008 surface expression was similarly analysed by flow cytometry. One clone, clone 3 was selected for FISH and confocal microscopy analysis.

### Protein expression

The DNA sequence encoding amino acid # 73-739 (DBL1α-CIDR1α: … CELDYRF…DTKTNPC), amino acid #473-817 (CIDR1α*: DYCQICP:… NGEPCTG), amino acid # 407-798 (CIDR1α: KDAKTDS…TLNGDIC), amino acid # 1239-1689 (DBL3β: CAETGGV… YATACDC), amino acid #1719-2255 (DBL4γ: PRDKTTG…LKGDKSL), amino acid #2258-2764 (DBL5δ: ACALKYG… SAKQKDC), and amino acid # 2242-3016 (DBL5δ-CIDR2β: CATVAKA…VTQPNIC) of 3D7 *PFD1235w*, amino acid #1553-1924 (CIDR2β: KKQEKLY…NVPANPC) and amino acid #1994-2378 (DBL4β: CNITKEH… HDDACAC) of 3D7 PF11_0008 and the DNA amplified by primers listed in Table S1 was cloned and expressed in a baculovirus system as described [39], [77]–[79]. DBL1x VAR2CSA protein was kindly provided by Ali Salanti and Madeleine Dahlbäck.

### Generation of antisera

All procedures complied with European or national regulations. Prior to immunization each animal was pre-bled and these sera were used as negative controls in the flow cytometry and confocal microscopy experiments. VAR2CSA DBL5ε-DBL6ε rat antisera, rabbit αDBL5ε-DBL6ε and mouse αDBL5ε antisera [80] were kindly provided by Ali Salanti and Madeleine Dahlbäck. PFD1235w-CIDR1α,PFD1235w-DBL4γ, and PF11_0008-CIDR2β rabbit, and PFD1235w-DBL1α-CIDR1α, PFD1235w-CIDR1α^*^, PFD1235w-DBL3β, PFD1235w-DBL4γ, PFD1235w-DBL5δ, PFD1235w-DBL5δ-CIDR2β, and PF11_0008-DBL4β were raised by subcutaneous injection of 10–20 µg protein in complete Freund's adjuvant followed by several boosters of protein in incomplete Freund's adjuvant.

### Ethics statement

All experiments including immunizations and bleeding of animals was approved by The Danish Animal Procedures Committee (“Dyreforsoegstilsynet”) as described in permit no. 2008/561-1498 and according to the guidelines described in act no. LBK 1306 (23/11/2007) and BEK 1273 (12/12/2005).

### Affinity purification of antibodies

0.5 mg of PFD1235w-DBL4γ and PF11_0008-CIDR2β in each 1 ml were dialysed ON against coupling buffer and subsequently coupled to HiTrap NHS activated HP columns as described by the manufacturer (GE Healthcare). A pool of 3 ml PFD1235w-DBL4γ antisera from six rats and 6 ml PF11_0008-CIDR2β antisera from one rabbit were diluted 1∶1 in PBS and affinity purified on the HiTrap columns. Following elution in Glycin buffer (0.1 M, pH 2.8) antibodies were neutralised in Hepes buffer (1M, pH 8.0).

### Depletion and blocking of antibodies

The affinity purified rabbit PFD1235w-DBL4γ and PF11_0008-CIDR2β antibodies were depleted for V5-His reactivity using a non-sense peptide (VLIM-tag) VLIMFNEQHKRGASTYWCPGKPIPNPLLGLD STRTGHHHHHH (Schafer-N) containing the V5-His epitope (underlined) and recombinant V5-HIS tagged VAR2CSA-DBL1x. The VLIM-tag peptide and VAR2CSA-DBL1x (both 1 mg/ml in PBS) were coupled to Epoxy M270 Dynabeads according to the manufacturer's instructions (Invitrogen). 200 µl of each of the two different species of affinity purified antibodies was incubated for 2 h at RT with 3 mg of coupled Dynabeads. Following this the supernatant containing the depleted antibody fraction was removed and re-incubated twice with freshly coupled Dynabeads. Antibodies bound to the Dynabeads were eluted in Glycin/HCl (0.1 M, pH 2.75) and neutralised in Tris buffer (1 M, pH 9.0). The reactivity of depleted and non-depleted antibody fractions was tested by ELISA (as described below) using PFD1235w-DBL4γ, PF11_0008-CIDR2β, VAR2CSA-DBL1x, and the VLIM-tag peptide as coating antigen. Additionally, the affinity purified and depleted PFD1235w-DBL4γ and PF11_0008-CIDR2β antibodies used for flow cytometry and confocal microscopy were tested by Luminex (as described below) on 49 different PfEMP1 recombinant proteins. Similarly rabbit antisera against PFD1235w-CIDR1α was depleted on recombinant V5-HIS-tagged VAR2CSA-DBL1x and tested by ELISA using PFD1235w-CIDR1α, PF08_0103-CIDR1α, PF11_0008-CIDR2β, VAR2CSA-DBL1x, and the VLIM-tag peptide as coating antigen.

Rat PFD1235w-DBL4γ, rabbit PFD1235w-CIDR1α or PF11_0008-CIDR2β antisera binding was blocked by incubation for 1 hr at 4°C using excess purified recombinant PFD1235w-DBL4γ, PFD1235w-CIDR1α, PF11_0008-CIDR2β, or PF08_0103-CIDR1α protein (3 µg/well containing 5 µl rat or 10 µl rabbit sera) and remaining cross-reactivity to parasite antigens expressed on the surface of infected erythrocytes was subsequently tested by flow cytometry as described below.

### Flow cytometry

Parasite cultures were repeatedly antibody selected as described above and grown for 3–5 cycles prior to doing flow cytometry. Single colour flow cytometry surface staining was done with minor modifications as described [74]. In brief, IE were purified on a magnet-activated cell sorting column (MACS) and 2×10^5^ ethidium bromide-labelled IE were incubated for 30 min at 4°C in 5 µl rat, 10 µl rabbit sera, or 15 µl affinity purified and tag-depleted antibody depleted of anti-human erythrocyte antibodies and then incubated for 30 min at 4°C with FITC-conjugated goat-anti-rat IgG (1∶150, Zymed) or FITC-conjugated goat-anti-rabbit IgG (1∶200, Vector Laboratories). Prior to doing two colour flow cytometry surface staining the purity of the MACS-purified IE was verified by running a small sample of ethidium bromide-labelled IE on a flow cytometer. Sub-line MACS preparations with ≥90% IE were used for two colour flow cytometry. In the case of clone 3 MACS preparations with 75–80% IE were used. In brief, 2×10^5^ MACS-purified unlabelled IE were incubated with 5 µl rat and 10 µl rabbit sera depleted of anti-human erythrocyte antibodies, followed by incubation with FITC-conjugated goat-anti-rat IgG (1∶150, Zymed) and Alexa Fluor 610-R-PE-conjugated goat-anti-rabbit IgG (1∶200, Molecular Probes). Samples were analysed on a Cytomics FC 500 MPL flow cytometer (Beckman Coulter) and data analysed using WinList version 6.0 (Verity Software House Inc.). IE stained with one surface colour was gated based on the ethidium bromide staining to exclude uninfected erythrocytes. Two colour stained non-ethidium bromide-labelled IE were gated based on forward and side scatter values since ethidium bromide could not be used due to overlapping spectra with the Alexa Fluor 610-R-PE-conjugated anti-rabbit used. For removal of surface PfEMP1 and analysis of antisera cross-reactivity with trypsin resistant surface IE antigens expressed on late-stage trophozoites and schizonts IE were treated with 1 g/l porcine trypsin and 0.2 g/l EDTA solution in Hanks balanced salt solution (Sigma-Aldrich) for 10 min at 37°C. The reaction was stopped by adding 10% foetal calf serum (FCS) and cells washed three times in PBS plus 2% FCS. Controls were incubated similarly in PBS plus 2% FCS or in a trypsin/EDTA solution containing 10% FCS.

### Confocal microscopy

Laser scanning confocal microscopy was performed on all samples analysed by flow cytometry in order to observe the staining patterns on individual IE. MACS purified unlabelled infected cells from the same batches of parasites tested by flow cytometry were incubated with individual antibodies or in combination with the PFD1235w, PF11_0008, and VAR2CSA antibodies as previously described [81]. Briefly, 1 µl packed IE were washed in 1% BSA in PBS (BSA/PBS) and the pellet was incubated in 100 µl BSA/PBS and 3 µl of the respective antibodies for 30 minutes at 4°C. The IE were washed three times in BSA/PBS. The IE stained with primary antibody were then incubated with secondary antibodies, either Alexa 488 anti-rat IgG (Invitrogen), Alexa 568 anti-rabbit and/or 568 Alexa anti-mouse IgG (Invitrogen) and DAPI (3 µl of a 3 µg/ml solution) for 30 minutes at 4°C. The IE were washed three times and visualised as live, unfixed cells using a Nikon TE 2000-E confocal Nikon microscope with 60x oil immersion objective lens (DIC). The images were processed using Adobe Photoshop software and displayed with the 5 µm scale bar calculated by the EZ-C1 software.

### ELISA and luminex

Affinity purified and depleted rat and rabbit antibodies were tested by ELISA. Wells of Maxisorp plates (Nunc, Roskilde, Denmark) were coated with 1 µg/ml of testing antigen in Glycin/HCl buffer (0.1 M, pH 2.75), antibodies were diluted 1∶100 in blocking buffer (PBS, 0.5 M NaCl, 1% Triton-X-100, 1% BSA, pH 7.2), and plates were washed and developed as described previously [77]. In addition, the affinity purified and depleted antibodies were tested in the BioPlex^100^ System (BioRad) as previously described [40]. Briefly, 0.1 mg Baculovirus produced proteins and the VLIM-tag peptide were individually coupled to 1.25•10^7^ Luminex xMAP technology microsphere beads (Ramcon). Plex 1 and 2 contained 6 and 45 different proteins, respectively. Three protein domains were coupled twice in plex 2 and two were identical to domains in plex 1 (Table S1). Prior to multiplexing, protein coupling was verified by incubating beads (1∶333) with mouse anti-V5 antibody (1∶10,000, Invitrogen) followed by biotinylated anti-mouse IgG (1∶500, DakoCytomation). The biotinylated antibody was detected using PE-streptavidin (1∶500, Sigma) and diluents used were PBS/TB (PBS, 0.05% (v/v) Tween-20, 0.1% (w/v) BSA, pH 7.4). Multiplexed beads (1∶333) were incubated with rat or rabbit antibodies (1∶1000) followed by incubation with biotinylated anti-rat IgG (Sigma) or anti-rabbit IgG antibody (The Binding Site) diluted 1∶1000. Detection was done with PE-streptavidine 1∶100 or 1∶1000 for rat and rabbit antibodies, respectively. Beads were resuspended in 100 µl diluents and a minimum of 100 beads from each set of multiplexed beads were analyzed to yield the mean fluorescence intensity (MFI). Each affinity purified and depleted antibody was analyzed in duplicates, the average MFIs and standard deviation were calculated based on three independently repeated experiments.

### RNA extraction and cDNA synthesis

Antibody selected IE were grown for 3–5 cycles and erythrocytes infected by trophozoite/schizont-stage parasites (20–48 h post invasion) from *in vitro* cultures were MACS purified and used for flow cytometry, confocal microscopy and for re-invasion of uninfected erythrocytes in new cultures flasks. IE used for RNA extraction were harvested when re-invaded parasites were at the ring-stage as confirmed by microscopy of Giemsa stained thin smears.

Total RNA was prepared using Trizol (Invitrogen) as recommended by the manufacturers and treated with DNase1 (Invitrogen) for 15 min at 37°C. Absence of DNA in RNA samples was confirmed by stable base fluorescence after 40 cycles of real-time PCR with *seryl-tRNA synthetase* and *fructose-bisphosphate aldolase* primers as previously described [41]. Superscript II was used to reverse transcribe DNA-free RNA primed with random hexamer primers (Invitrogen) at 25°C for 10 min and 42 C for 50 min followed by 70°C for 15 min.

### Quantitative real-time PCR

Quantitative real-time PCR was performed using a Rotorgene thermal cycler system (Corbett Research), Quanti-Tect SYBR Green PCR Master Mix (QIAGEN), and real-time PCR-optimized and gene-specific primers (0.5 µM) for each of the full-length *var* genes in the *P. falciparum* 3D7 genome and to the endogenous control genes *seryl-tRNA synthetase* and *fructose-bisphosphate aldolase*. Quantification was done using Rotorgene software version 6.0. mRNA transcript copy numbers were calculated for each *var* transcript using primer pair specific standard curves generated from real-time Q-PCR measurements of 10 fold genomic DNA dilutions as described [43]. The proportions of individual *var* gene transcripts relative to the total copy number of all *var* transcripts or to the total number of endogenous control genes was subsequently calculated for comparison of *var* transcript profiles within and between samples and depicted in pie-charts.

### FISH analysis

RNA-FISH was done on ring stage parasites of 3D7_PFD1235w_, 3D7_PF11_0008_, and 3D7_PFD1235w/PF11_0008_ IE. PCR products were amplified using 3D7 genomic DNA and *PFD1235w* (fw: 5′-GGGATCCGACACGTCGAGACAGAGG-′3; rv: 5′-GGAGAAGCTTTGCAGCGGACTTCACA-′3) and *PF11_0008* (fw: 5′-GGGATCCTAGTTATTTGACGCACCAGC-′3 and 5′-CGTGAATTCGGTGGCTGTACCTTCCC-3′) specific primers containing restriction sites (underlined). The *PFD1235w* and *PF11_0008* PCR products were subsequently cloned into the pSPT19 or pSPT18 vector (Roche Applied Science), respectively and sequenced on a 3130 Genetic Analyzer (AppliedBiosystems). Digoxigenin (DIG)- and biotin-labelled antisense RNA probes were generated using a DIG RNA Labelling Kit and Biotin RNA Labelling Mix (Roche Applied Science). The *var2CSA* anti-sense probe described previously [50] was included as a negative control. The specificity of the RNA probes was confirmed by standard Northern blotting analysis according to the DIG Application Manual found at http://www.roche-applied-science.com/PROD_INF/MANUALS/DIG_MAN/dig_toc.htm and [39].

The RNA-FISH slides were prepared according to a standard FISH protocol with minor modifications [82], [83]. Following hybridization over-night at 48°C, single stained anti-sense RNA were detected by α-DIG HRP conjugated Ab or α-biotin HRP conjugated Ab and the dual stained anti-sense RNA were detected with both antibodies. The signal was further detected using the TSA Plus Fluorscence Palette System (PerkinElmer) with FITC and/or Cyanine 3, according to the protocol as described [83]. As control, slides were treated with RNAse (Sigma-Aldrich) at a concentration of 100 µg/ml for 30 min at 37°C prior to hybridization with the probes. Slides were washed, mounted with anti-fade reagent containing DAPI (Invitrogen) and images captured using a Nikon TE 2000-E confocal microscope as described above. The positivity of a total number of 100 IE was scored in each experiment.

### Intron real-time PCR and sequencing of intron regions

RNA was isolated from the 3D7_PFD1235w_, 3D7_PF11_0008_, and 3D7_PFD1235w/PF11_0008_ sub-lines and cDNA generated as described above. Real-time quantitative PCR was done using primers amplifying the intron spanning region and parts of exon 1 of the *var* genes listed in Table S2 as well as the *seryl-tRNA synthetase* and *fructose bisphosphate aldolase* [41]. In addition, PCR products were amplified from cDNA using TaKaRA La Taq (Lonza) and the intron spanning primers of *PFD1235w* and *PF11_0008* and the *var* genes listed in Table S2. The PCR products were sequenced on a 3130 Genetic Analyzer using the same set of primers. For comparison genomic DNA was purified from a batch culture of 3D7_PFD1235w/PF11_0008_ as well as from clone 3 and the intron spanning region of PFD1235w and PF11_0008 was sequenced.

### Adhesion assays

Adhesion assays were done as described previously [84]. In brief, ring stage IE were cultured over night in hypoxanthine-free RPMI 1640 (Lonza) with 10% hypoxanthine-free Albumax (Invitrogen) and 8.75 MBq^ 3^H-hypoxanthine (Amersham) per ml packed erythrocytes. 96-well flat bottomed plates (Nunc) were coated with 1% gelatine and HUVEC cells (PromoCell) grown in endothelial cell growth medium (Promocell) to maximum 6^th^ passage were seeded at 550,000 cells per well and grown to become confluent. CHO cells (CHO-wild type, CHO-CD36, CHO-CD54/ICAM1 from ATCC) were grown in RPMI 1640 with 1% added glutamine and 10% foetal calf serum (Lonza) and similarly seeded at 550,000 cells per well. On the day of the assay, ^3^H-labelled parasite cultures were enriched for late-stages by gelatine floatation and 50 µl of labelled trophozoites (12,000 counts per minute) were added in duplicates or triplicates to the 96-well plates containing HUVEC or CHO cells. The plates were left to incubate on a rocking table for 2 hrs at 37°C. Monoclonal mouse anti-CD54/ICAM1 My13 (10 µg/ml, Invitrogen), anti-CD54/ICAM1 15.2 (20 µg/ml, AbD Serotec), anti-CD36 (10 µg/ml, R&D) and anti-CD31/PECAM-1 9G11 (20 µg/ml, R&D Systems) was added in anti-adhesion assays. Unbound IE were removed using a washing robot (Biomek 2000, Beckman Coulter) and cells binding IE were harvested onto filter paper (Unifilter-96, GF/C, PerkinElmer) using a Filtermate Harvester (PerkinElmer). Following addition of 50 µl/well of scintillation liquid (Microscint-20, PerkinElmer) counting was done on a Topcount NXT (Perkin-Elmer). The ^3^H max-values were determined as the counts per minute of 50 µl of parasite added to one cell-free well and harvested directly onto a filter paper. To standardise, all values obtained was given as a ratio compared to the max value obtained in the particular assay. The percentage binding of the different 3D7 sub-lines were calculated relative to the binding of the 3D7_PFD1235w_ parasite line adhering to CD54/ICAM1 (Figure 6A–C) or the 3D7_PF11_0008_ parasite line adhering to HUVEC (Figure 6D) (e.g. binding of these equalling a 100%).

### Accession numbers

Nucleotide sequence and protein data reported in this paper are available from http://plasmodb.org/plasmo/under accession numbers (accession numbers of data available from NCBI are shown in brackets): MAL6P1.1/PFF1595c (XP_966307), MAL6P1.314 (XP_965999), MAL6P1.316/PFF0010w (XP_965997), MAL6P1.4/PFF1580c (XP_966305), PF07_0049 (XP_001349031), PF07_0050 (XP_001349033), PF07_0051 (XP_001349034), PF07_0073 (XP_001349080) (seryl-tRNA synthetase), PF08_0103 (XP_001349434), PF08_0140 (XP_001349512), PF08_0141 (XP_001349513), PF11_0008 (XP_001347692), PF11_0521 (XP_001348176), PF13_0003 (XP_001349740), PFA0015c (XP_001350938), PFC0005w (XP_001351080), PFD0005w (XP_001351319.), PFD0020c (XP_001351321), PFD0615c (XP_001351435), PFD0625c (XP_001351437), PFD1235w (XP_001351561), PFE1640w (XP_001351876), PFI1820w (XP_001352240), PFL0005w (XP_001350410), PFL0020w (XP_001350413), PFL0030c (XP_001350415) (VAR2CSA), PFL1955w (XP_001350797), and PFL2665c (XP_001350935).

## Supporting Information

Figure S1 PfEMP1 surface expression on 3D7 infected erythrocytes and antisera specificity. (A) erythrocytes infected with the 3D7_PFD1235w_, (B) 3D7_PF11_0008_, and (C) 3D7_PFD1235w/PF11_0008_ sub-line. Surface staining of PfEMP1 by flow cytometry was done using rat antisera against (1) DBL1-CIDR1α, (2) CIDR1α*, (4) DBL3β, (5) DBL4γ, (7) DBL5δ and (8) DBL5δ-CIDR2β of PFD1235w and (9) DBL4β of PF11_0008 as well as using rabbit antisera against (3) CIDR1α of PFD1235w, (6) DBL4γ of PFD1235w, and (10) CIDR2β of PF11_0008. Flow cytometry settings were identical for all panels and reactivity with pre-bleeds is shown as grey histograms
(0.11 MB DOC)

Figure S2 Trypsination of 3D7 infected erythrocytes and blocking of antisera show no cross-reactivity. (A) and (D) 3D7_PFD1235w_ IE, (B) and (E) 3D7_PF11_0008_ IE, (C) and (F) 3D7_PFD1235w/Pf11_0008_ IE. (A-C) Untreated (light grey bars), trypsin (black bars), and trypsin plus foetal calf serum (FCS) treated IE (dark grey bars) were surface stained for flow cytometry using rabbit sera against PFD1235w-DBL4γ, PFD1235w-CIDR1α, PF11_0008-CIDR2β and rat sera against PFD1235w-DBL4γ (*). (D-F) Rabbit sera against PFD1235w-CIDR1α and PF11_0008-CIDR2β and rat sera against PFD1235w-DBL4γ (see vertical lines) were blocked prior to flow cytometry with recombinant PFD1235w-CIDR1α or DBL4γ, PF11_0008-CIDR2β or PF08_0103-CIDR1α as indicated. (A-F) Relative MFI values were calculated as MFI_sample_/MFI_pre-bleed_. The dotted line indicates the MFI_pre-bleed_/MFI_pre-bleed_ value.
(0.28 MB DOC)

Figure S3 PfEMP1 surface expression on erythrocytes infected with 3D7_PFD1235w/PF11_0008_ clone 3 and 4. Single colour surface staining of PfEMP1 by flow cytometry was done using (A) rabbit pre-bleed (1), αPFD1235w-DBL4γ (2), αPF11_0008-CIDR2β (3) antisera, and (B) rat pre-bleed (1), αPFD1235wDBL4 γ (2), αPFD1235w-DBL5δ-CIDR2β (3), and αPF11_0008-DBL4β (4) antisera. (C) Double colour surface staining of PfEMP1 by flow cytometry was done using (1) buffer, (2) rabbit and rat pre-bleed, (3) rat αPFD1235w-DBL4γ and rabbit αPF11_0008-CIDR2β, (4) rabbit αPFD1235w-DBL4γ and rat αPF11_0008-DBL4β, (5) rat αPFD1235w-DBL4γ and rabbit pre-bleed, (6) rabbit and αPF11_0008-DBL4β rat pre-bleed, and (7) rat αPFD1235w-DBL5δ-CIDR2β and rabbit αPF11_0008-CIDR2β. Flow cytometry settings were identical for all panels and reactivity with buffer in (A) and (B) is shown as grey histograms. Number inserts are percentage positive cells.
(0.14 MB PDF)

Figure S4 The specificity of antisera and antibodies used for surface labelling and selection of parasites. The specificity was tested by ELISA (A-C) and by Luminex (D-F) on 49 different PfEMP1 domains (Table S1). (A) rat PFD1235w-DBL4γ antisera, (B) rabbit PF11_0008-CIDR2β antisera, (C) rabbit FD1235w-CIDR1α antisera, (D) antibodies purified on a PFD1235w-DBL4γ column and depleted of tag reactivity, (E) antibodies purified on a PF11_0008-CIDR2β column and depleted of tag reactivity, and (F) PFD1235w-CIDR1α antisera depleted of tag reactivity. The coating antigens in ELISA were DBL4γ and CIDRα of PFD1235w, CIDR1α of PF08_0103, CIDR2β of PF11_0008, DBL1x of VAR2CSA, and the VLIM-tag peptide as indicated by the vertical lines in (A-C).
(0.02 MB PDF)

Figure S5 Simultaneous surface expression of PfEMP1 on single erythrocytes infected with 3D7 detected using purified antibodies. Parasite cultures (A-D) were identical to those used in Figure 1, Figure 2, and Figure S1. The affinity purified and depleted antibodies used were (A-D1) rat αPFD1235w-DBL4γ; (A-D2) rabbit PF11_0008-CIDR2β antibodies; and (A-D3), rat αPFD1235w-DBL4γ combined with rabbit αPF11_0008-CIDR2β. For confocal microscopy rat antibody staining of PFD1235w expressed by the 3D7_PFD1235w_ (A1 & A3) and 3D7_PFD1235w/PF11_0008_ (C1 & C3) sub-lines were detected using Alexa 488-labeled anti-rat antibodies (green) and rabbit antibodies staining PF11_0008 expressed by the 3D7_PF11_0008_ (B2) and 3D7_PFD1235w/PF11_0008_ (C2 & C3) were detected using Alexa 568-labeled anti-rabbit antibodies (red). Double staining using the two Alexa fluorophores (A-D3) showed simultaneous expression of PFD1235w and PF11_0008 by RBC infected with the 3D7_PFD1235w/PF11_0008_ with no co-localisation of the staining (C3). Flow cytometry histograms show single staining of the three different IE sub-lines using the purified and depleted antibodies. Double staining was not done due to limited amounts of depleted antibody. (D1-3) A NF54_VAR2CSA_ control sub-line did not stain positive with any of the antibody preparations. DAPI staining of DNA in the nuclei is blue. Scale bar 5 μm.
(0.06 MB PDF)

Figure S6 Real-time Q-PCR Ct-values for exon I and intron crossing amplicons amplified from cDNA of 3D7. (A) 3D7_PFD1235w_ sub-line. (B) 3D7_PF11_0008_ sub-line. (C) 3D7_PFD1235w/PF11_0008_ sub-line. Numbers on the Y-axis are primer numbers (See Table S2). Grey bars: exon I primers. Black bars: intron primers. White bars: house keeping gene primers.
(0.01 MB PDF)

Table S1 Primers used for amplification of DNA encoding recombinant proteins coupled to Luminex beads.
(0.09 MB DOC)

Table S2 Specific *var* gene primers used for *var* intron Q-RT PCR.
(0.05 MB DOC)

Video S1 Simultaneous surface expression of PfEMP1 on single erythrocytes infected with *Plasmodium falciparum* 3D7_PFD1235w/PF11_0008_. Staining of PFD1235w and PF11_0008 is green and red, respectively. DAPI staining of DNA in the nuclei is blue. Scale bar 5 μm.
(2.30 MB MOV)
