# Supplementary figures and images for: Surface Co-Expression of Two Different PfEMP1 Antigens on Single Plasmodium falciparum-Infected Erythrocytes Facilitates Binding to ICAM1 and PECAM1
Source: PLoS Pathog. 2010 Sep 2;6(9):e1001083. doi: 10.1371/journal.ppat.1001083 (PMC2932717; doi:10.1371/journal.ppat.1001083)

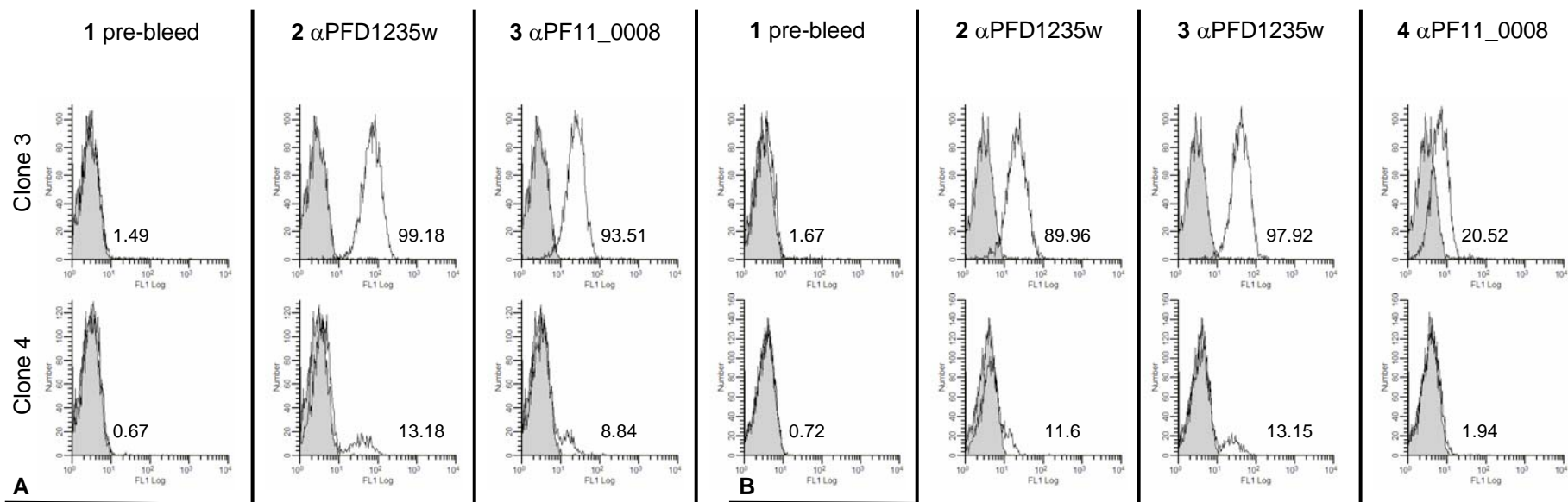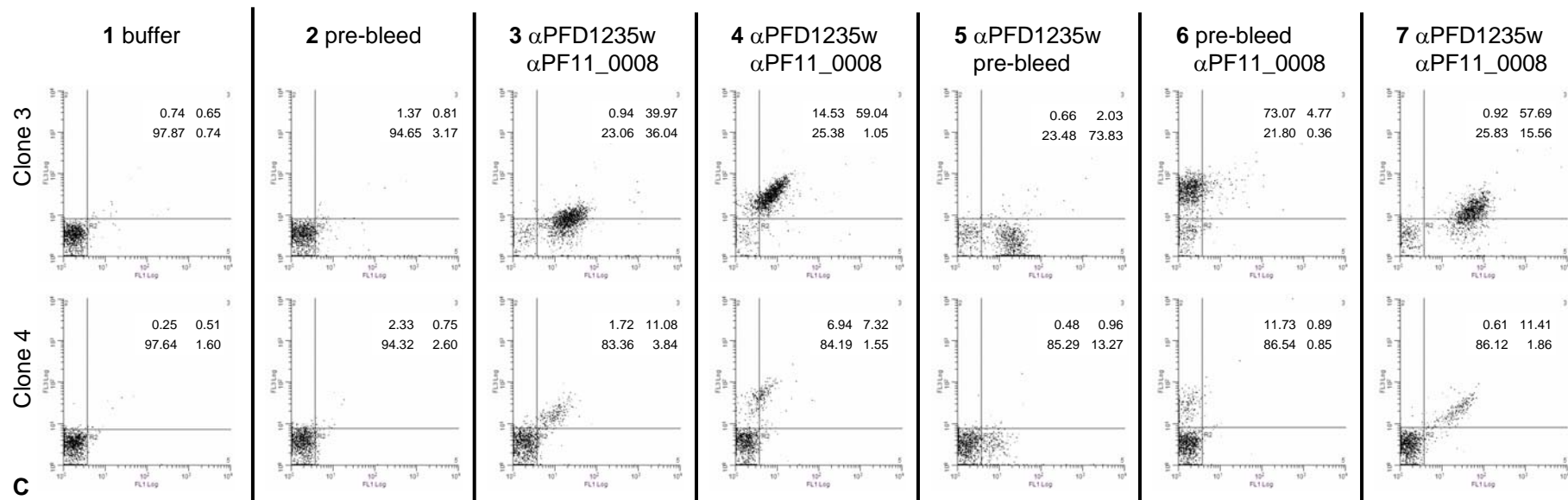

Supplement: Figure S3 — PfEMP1 surface expression on erythrocytes infected with 3D7PFD1235w/PF11_0008 clone 3 and 4. Single colour surface staining of PfEMP1 by flow cytometry was done using (A) rabbit pre-bleed (1), αPFD1235w-DBL4γ (2), αPF11_0008-CIDR2β (3) antisera, and (B) rat pre-bleed (1), αPFD1235wDBL4 γ (2), αPFD1235w-DBL5δ-CIDR2β (3), and αPF11_0008-DBL4β (4) antisera. (C) Double colour surface staining of PfEMP1 by flow cytometry was done using (1) buffer, (2) rabbit and rat pre-bleed, (3) rat αPFD1235w-DBL4γ and rabbit αPF11_0008-CIDR2β, (4) rabbit αPFD1235w-DBL4γ and rat αPF11_0008-DBL4β, (5) rat αPFD1235w-DBL4γ and rabbit pre-bleed, (6) rabbit and αPF11_0008-DBL4β rat pre-bleed, and (7) rat αPFD1235w-DBL5δ-CIDR2β and rabbit αPF11_0008-CIDR2β. Flow cytometry settings were identical for all panels and reactivity with buffer in (A) and (B) is shown as grey histograms. Number inserts are percentage positive cells. (0.14 MB PDF) [file ppat.1001083.s003.pdf]

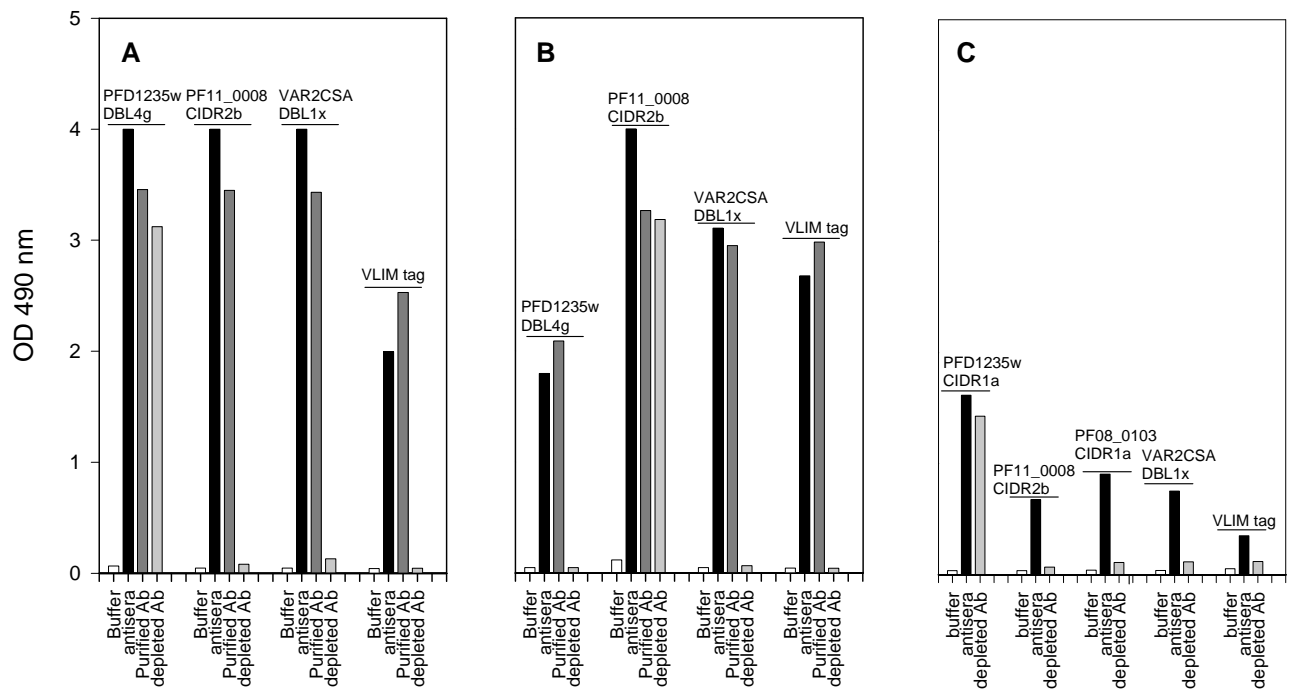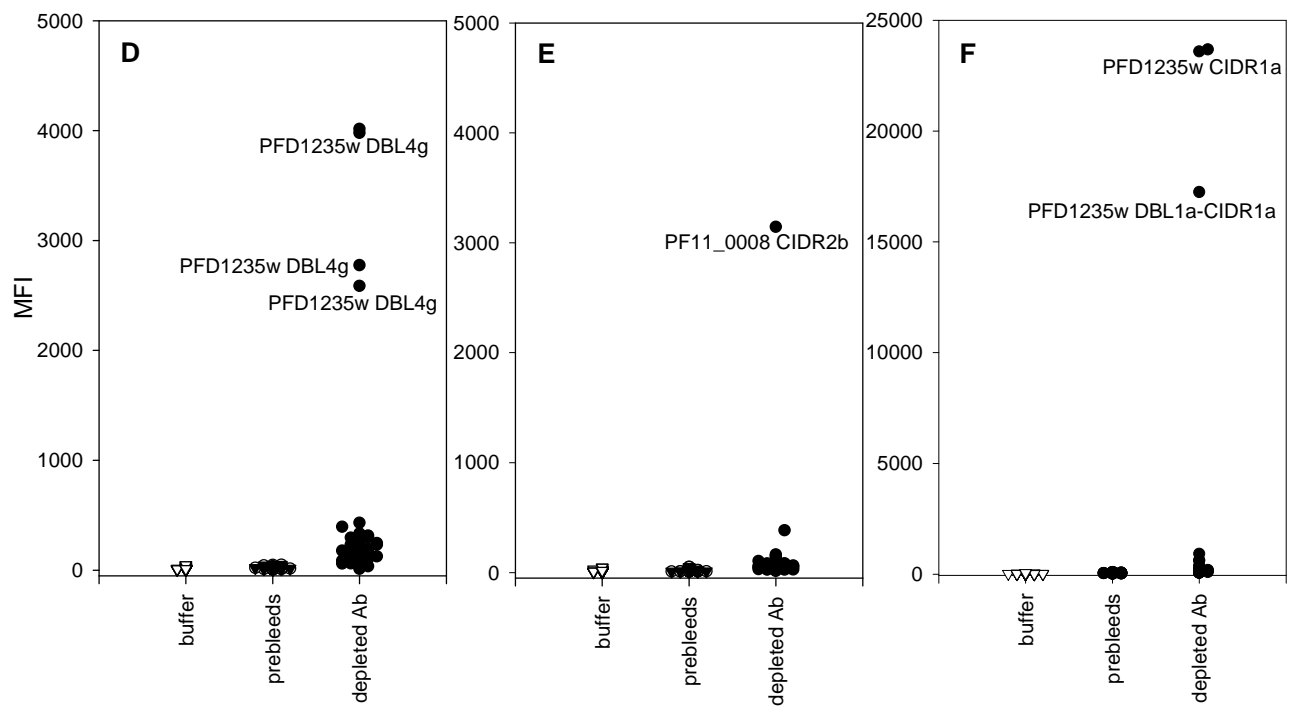

Supplement: Figure S4 — The specificity of antisera and antibodies used for surface labelling and selection of parasites. The specificity was tested by ELISA (A-C) and by Luminex (D-F) on 49 different PfEMP1 domains (Table S1). (A) rat PFD1235w-DBL4γ antisera, (B) rabbit PF11_0008-CIDR2β antisera, (C) rabbit FD1235w-CIDR1α antisera, (D) antibodies purified on a PFD1235w-DBL4γ column and depleted of tag reactivity, (E) antibodies purified on a PF11_0008-CIDR2β column and depleted of tag reactivity, and (F) PFD1235w-CIDR1α antisera depleted of tag reactivity. The coating antigens in ELISA were DBL4γ and CIDRα of PFD1235w, CIDR1α of PF08_0103, CIDR2β of PF11_0008, DBL1x of VAR2CSA, and the VLIM-tag peptide as indicated by the vertical lines in (A-C). (0.02 MB PDF) [file ppat.1001083.s004.pdf]

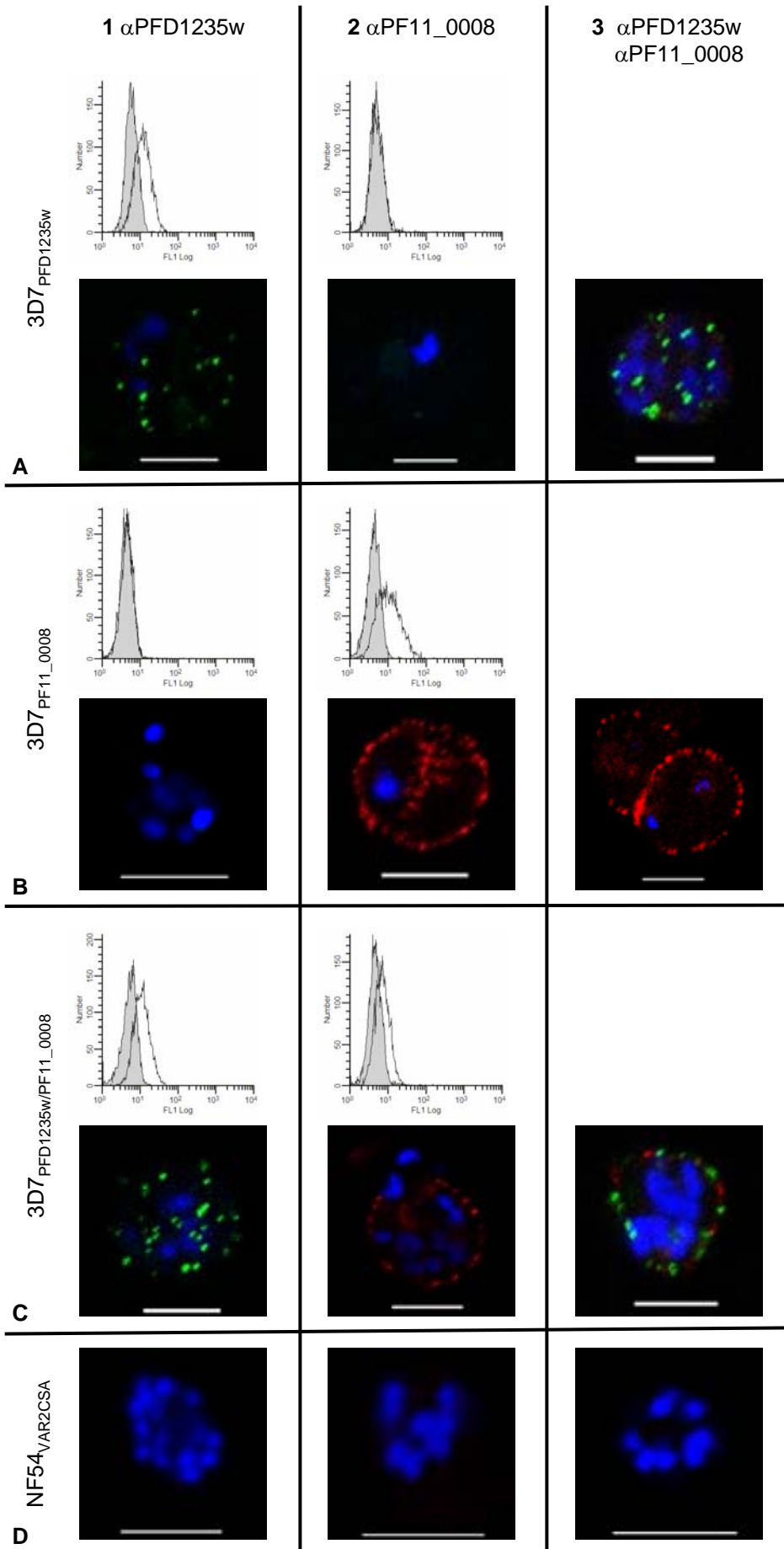

Supplement: Figure S5 — Simultaneous surface expression of PfEMP1 on single erythrocytes infected with 3D7 detected using purified antibodies. Parasite cultures (A-D) were identical to those used in Figure 1, Figure 2, and Figure S1. The affinity purified and depleted antibodies used were (A-D1) rat αPFD1235w-DBL4γ; (A-D2) rabbit PF11_0008-CIDR2β antibodies; and (A-D3), rat αPFD1235w-DBL4γ combined with rabbit αPF11_0008-CIDR2β. For confocal microscopy rat antibody staining of PFD1235w expressed by the 3D7PFD1235w (A1 & A3) and 3D7PFD1235w/PF11_0008 (C1 & C3) sub-lines were detected using Alexa 488-labeled anti-rat antibodies (green) and rabbit antibodies staining PF11_0008 expressed by the 3D7PF11_0008 (B2) and 3D7PFD1235w/PF11_0008 (C2 & C3) were detected using Alexa 568-labeled anti-rabbit antibodies (red). Double staining using the two Alexa fluorophores (A-D3) showed simultaneous expression of PFD1235w and PF11_0008 by RBC infected with the 3D7PFD1235w/PF11_0008 with no co-localisation of the staining (C3). Flow cytometry histograms show single staining of the three different IE sub-lines using the purified and depleted antibodies. Double staining was not done due to limited amounts of depleted antibody. (D1-3) A NF54VAR2CSA control sub-line did not stain positive with any of the antibody preparations. DAPI staining of DNA in the nuclei is blue. Scale bar 5 μm. (0.06 MB PDF) [file ppat.1001083.s005.pdf]

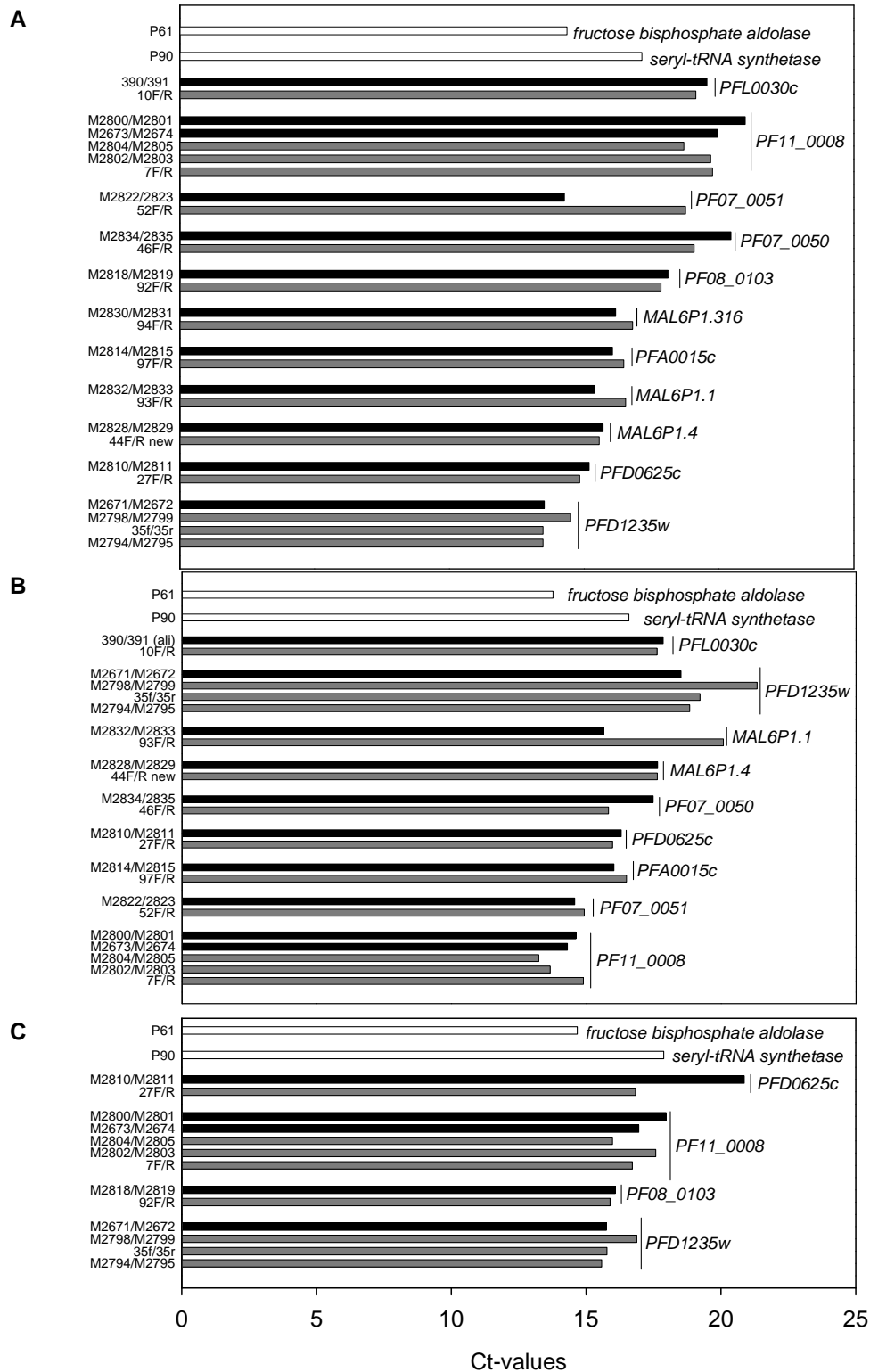

Supplement: Figure S6 — Real-time Q-PCR Ct-values for exon I and intron crossing amplicons amplified from cDNA of 3D7. (A) 3D7PFD1235w sub-line. (B) 3D7PF11_0008 sub-line. (C) 3D7PFD1235w/PF11_0008 sub-line. Numbers on the Y-axis are primer numbers (See Table S2). Grey bars: exon I primers. Black bars: intron primers. White bars: house keeping gene primers. (0.01 MB PDF) [file ppat.1001083.s006.pdf]
