# Supplementary material for: Surface Co-Expression of Two Different PfEMP1 Antigens on Single Plasmodium falciparum-Infected Erythrocytes Facilitates Binding to ICAM1 and PECAM1
Source: PLoS Pathog. 2010 Sep 2;6(9):e1001083. doi: 10.1371/journal.ppat.1001083 (PMC2932717; doi:10.1371/journal.ppat.1001083)
Supplement: Table S1 — Primers used for amplification of DNA encoding recombinant proteins coupled to Luminex beads. (0.09 MB DOC) [file ppat.1001083.s007.doc]

| Genome | PfEMP1_domain | Forward primer | Reverse primer |
| --- | --- | --- | --- |
| 3D7 | PFA0015C_whole^c^ | ctctagaatgtagtcttgatcacaaattcc | tgcggccgctgtatgatgcagcacattcctc |
| 3D7 | PFC0005w_CIDR1α^b,c^ | ΑCΑGGΑTCCTGΑTΑΑΑGΑΑΑTΑΑΑΑΑΑΑGCΑTΑTG | ΑCΑGCGGCCGCΑTGCTTCΑTTGΑGΑGCΑGTGTT |
| 3D7 | PFD0005w_DBL3δ^c^ | CTCTAGAATGTACCCTCAAATATGGC | TGCGGCCGCTATATGCACTTTTTTGTTCCCC |
| 3D7 | PFD0005w_CIDR1α^c^ | ΑΑCGΑΑTTCΑCΑΑΑΑGΑΑΑTΑΑCΑΑGTGGTGG | ΑCΑGCGGCCGCΑTTGCGGΑTCCTCTTCCTCCTCC |
| 3D7 | PFD0020c_DBL3γ^c^ | cggatccctgtaatggaattaagacacttcttg | TGCGGCCGCTcgcactttgtgttggtgctg |
| 3D7 | PFD0020c_DBL2β^c^ | CGGATCCCTGCAACATTGATACAAGCTATTC | TGCGGCCGCTATCGTTTTTTTTCTCAATTGGTTTTTG |
| 3D7 | PFD0615c_DBL2δ^c^   | ctctagaaTGCGGAACAAAATATGATAAATATG | tgcggccgctatttgttaccattatcacttgtagc |
| 3D7 | PFD1235w_NTS^a^ | CGGAATTCaaCGCAGAAGATAGAAAT | ATAAGAATgcggccgcTTGGTGTGCATGTTGTACCG |
| 3D7 | PFD1235w_ DBL1α-CIDR1α^a^ | CACCATGCCCGGGTGCGAGCTGGACTACCGCTTC | GAATTCGCAAGGGTTGGTCTTGGTGTCCACGG |
| 3D7 | PFD1235w_CIDR1α^a^ | CGgaattcGACTATTGCCAAATATGTC | ATAAGAATgcggccgcTACCTGTACATGGTTCACC |
| 3D7 | PFD1235w _DBL4γ^a^ | CGGAATTCACTACAGGTGGAATAGAT | ATAAGAATgcggccgcGTATACATTTCCAACCTAAACG |
| 3D7 | PFD1235w_DBL4γ^a^ | GAATTCACTACAGGTGGAATAGATCA | ACATGCGGCATTGAGACT |
| 3D7 | PFD1235w_  DBL5δ-CIDR2β^a^ | CACCATGCCCGGGTGCGCCACCGTGGCCAAG | GAATTCGCAGATGTTGGGCTGGGTCACAGGG |
| 3D7 | PFD1235w_DBL4γ^c^ | GAATTCACTACAGGTGGAATAGATCA | ACATGCGGCATTGAGACT |
| 3D7 | PFD1235w_CIDR1α^b,c^ | CGgaattcGACTATTGCCAAATATGTC | ATAAGAATgcggccgcTACCTGTACATGGTTCACC |
| 3D7 | PFE1640w_DBL2β^c^ | CTCTAGAATGTTATGGAAAAGATGGCAATAAG | TGCGGCCGCTCGGTTCCACTAGTACTATCCC |
| 3D7 | PFE1640w_DBL3γ^c^ | CTCTAGAATGCAATGTAGTGAATACACTAC | TGCGGCCGCTATGTTCCTTTGCATCATAGTAC |
| 3D7 | MAL6P1.1_CIDR1α^c^ | acaggatcctgaaaatgaaatgttaagtataagta | acagcggccgcatgcgacttctttaaattgttgg |
| 3D7 | MAL6P1.314_whole^c^ | ctctagaatgcgatcttgaacacagattc | tgcggccgctgtatgatgcagcacattcctc |
| 3D7 | MAL6P1.316_DBL4ζ^c^ | ctctagaaTGTGATAATAAATCAAGTAATCGCTTCAAC | tgcggccgctctataggtttttctgtgaaagcgtac |
| 3D7 | MAL6P1.316_DBL3γ^c^ | ctctagaaGTGTGTGAGATGGTGGACAC | tgcggccgctatgaatcactatttggtggaaaaacc |
| 3D7 | MAL6P1.4_CIDR2γ^c^ | cggatcccgttaacgaacatgaaaatgtaatatc | tgcggccgctcttcggttctttcttatcatc |
| 3D7 | MAL6P1.4_DBL4δ^c^ | CGGATCCCTGTTCAACAAAATATAAAAATGG | TGCGGCCGCTATATTTACTTTTTTGTTTATCATATTC |
| 3D7 | MAL6P1.4_DBL2β^c^ | CGGATCCCTGTAAAGGCAAAGATGGAAAC | TGCGGCCGCTCTCACACGCCTCATCATG |
| 3D7 | MAL6P1.4 _DBL6ε^c^ | CGGATCCCTGTGGTGATTTAAAATATAGC | TGCGGCCGCTTTCAGGATTCAATCCTTTG |
| 3D7 | MAL6P1.4_DBL7ε^b,c^ | CGGATCCCTGTCCTGAAGACATTGAATG | TGCGGCCGCTATATTCAGAGAGACAATGAAC |
| 3D7 | MAL6P1.4_DBL3γ^c^ | CGGATCCCTGTGAAAGTAGGCCACAAG | TGCGGCCGCTAGCTTCAGTAAATTTGTTATCATC |
| 3D7 | MAL6P1.4_DBL6ε^c^ | CGGATCCCTGTGGAAATTTTAGAACTC | CTGCGGCCGCTTATTCGTCGTTTTTTATATTTATTAC |
| 3D7 | PF07_0049_DBL2δ^c^ | CGGATCCCTGCCAACAAAAATATGCCAAAAAC | TGCGGCCGCTATATATACTTTTTTGATTTTGAAATTCTTC |
| 3D7 | PF08_0103_CIDR1α^c^ | ACAGAATTCGATAAAGAAAAAGAGAAAAACAATGAT | ACAGCGGCCGCAGGCGGCATTCAAGTCGCCTTTG |
| 3D7 | PF08_0140_CIDR1α^c^ | CTCGAATCCGGTGACCAAAAAGCAAAATG | CTGCGGCCGCTACAAGCGTCTTCATCCGCTG |
| 3D7 | PF08_0140_DBL2β^c^ | ctcgaatcctgtaacatagatgaaaaatattcc | tgcggccgctcgcctcttctttttttgttactg |
| 3D7 | PF08_0141_DBL4ζ^c^ | CGGATCCCTGTAAATTTAACGAAACATTTTG | TGCGGCCGCTTTCGTTATTATCAACAGGACATTC |
| 3D7 | PF08_0141_DBL2β^c^ | CGGATCCCTGTACAGGTAAAGATGGACAC | TGCGGCCGCTAAACTGAGTTTGTTTCTCAATTC |
| 3D7 | PF08_0141_DBL3γ^c^ | cggatccctgtaaaaacaaaaatggtgtc | TGCGGCCGCTtgtttctccattttgattggc |
| 3D7 | PFI1820w whole^c^ | CTCTAGAATGTAATCTTAGTCACAAATTCC | TGCGGCCGCTGTATGATGCAGCACATTCCTC |
| 3D7 | PF11_0008_CIDR2b^c^ | GAATTCAAAAAACAAGAAAAACTATAT | TGCGGCCGCTACATGGATTTGCTGGAACA |
| 3D7 | PF11_0008_DBL3δ^c^ | GAATTCGGATATGAAGATTATTCTG | TGCGGCCGCTAAATTCGTCTTTTTTTGTGTTTATC |
| 3D7 | PF11_0008_DBL2g^c^ | GAATTCTGTAATCCAAAAAAGGAT | TGCGGCCGCTTGGTTTATTCTGACTTTTATCAATATC |
| 3D7 | PF11_0521_CIDR1α^c^ | ACAGGATCCGCAAAGGAAATAGAAGCATATGTAAC | ACAGCGGCCGCATGGA TTTAGTGAATTACTGCGAG |
| 3D7 | PFL0005w_CIDR1α^c^ | acagaattcacaaatga aataaacaaaaagcat | acagcggccgcatgcatcttcaaaatttttggtgc |
| 3D7 | PFL0020w_DBL5ε^c^ | CTCTAGAATGCAAAAAATATGGTAGATATTCTTG | TGCGGCCGCTGTATTTTTCTATTATTTCATCAAAATTAC |
| 3D7 | PFL0020w_DBL4ζ^c^ | ctctagaatgtgataataaaggaaatgag | tgcggccgctttcgtccttgtcaacaggac |
| 3D7 | PFL1955w_CIDR1α ^c^ | cggatcccGATAAAGAAATAGAAAAATATAAAAACG | tgcggccgctatggcttctccgcattgtcttg |
| 3D7 | PFL1955w_CIDR1α^c^ | acagaattcgataaagaaatagaaaaatataaaaac | acagcggccgcatgcgacttctttaaattgttcg |
| 3D7 | PFL2665c_CIDR1α^c^ | ACAGΑΑTTCΑΑΑGGCGΑΑΑTΑΑCΑCGTGGT | ΑCΑGCGGCCGCΑGGCTTGTTTGΑG ΑTCGTCΑC |
| 3D7 | PF13_0003_CIDR1a^c^ | GAATTCGATAATAACGAAAATGGA | TGCGGCCGCTCTTATTATCACCATCAAC |
| FCR3 | VAR2CSA_CIDR1a^c^ | cggatccccaaagtaattctcatagtacatatg | tgcggccgctatggcccttgataagtataagc |
| FCR3 | VAR2CSA__DBL5e^c^ | cccccgggagatgttttgatgatcagaca | atttgcggccgccattacctttatcatactc |
| FCR3 | IT4var21_CIDR1α^c^ | ctctagaaacaaaagaaatatcagatggtgg | tgcggccgctacgcatcttggagactttcattg |
| HB3 | HB3var22_DBL3ε^c^ | cggatccctgtaaggatgaacaggagccg | tgcggccgctatgtcaaggcatcttttttcataaattcagg |
