# Supplementary material for: Surface Co-Expression of Two Different PfEMP1 Antigens on Single Plasmodium falciparum-Infected Erythrocytes Facilitates Binding to ICAM1 and PECAM1
Source: PLoS Pathog. 2010 Sep 2;6(9):e1001083. doi: 10.1371/journal.ppat.1001083 (PMC2932717; doi:10.1371/journal.ppat.1001083)
Supplement: Table S2 — Specific var gene primers used for var intron Q-RT PCR. (0.05 MB DOC) [file ppat.1001083.s008.doc]

| Primer set | Forward primer | Reverse primer | Target gene |
| --- | --- | --- | --- |
| 97f/97r | TCATTATGGGAAGCACGATT | TGATTTCTACCATCGCAAGG | *PFA0015c* |
| M2814/M2815^a^ | CATTATGGTTCCTGCTGGTATTG | CGTTGACTCGCATATGGGAT | *PFA0015c* |
| 27f/27r | TAAAAGACGCCAACAGATGC | TCATCGTCTTCGTCTTCGTC | *PFD0625c* |
| M2810/M2811^a^ | ACCGCCCTGGTGACATCCAC | CGATCACTTGCATAGGGGAT | *PFD0625c* |
| M2794/M2795 | GCATGTGCACCGTATAGGAG | CTATCACGACGATACAGATC | *PFD1235w* |
| 35f/35r | AAACACGTTGAATGGCGATA | GACGCCGAGGAGGTAAATAG | *PFD1235w* |
| M2798/M2799 | GGATCCACATGTATAAGAAG | TGATCTGGAAGTGCCATTAC | *PFD1235w* |
| M2671/M2672^a^ | GAAGATGAAGCTGTTACAGAATCCTTATCA | TTTCGGGGTAGGTGTTCCATAATC | *PFD1235w* |
| 93f/93r | GACAAATACGGCGACTACGA | TGTTTCACCCCATTCTTCAA | *MAL6P1.1/PFF1595c* |
| M2832/M2833^a^ | CAACCATCATGTGGAGTATTG | GTGTAACCACTATCTGTTCCAC | *MAL6P1.1/PFF1595c* |
| 94f/94r | TGGAAAGAACATGGACCTGA | TTCCTCGAGGGAAGAATCAC | *MAL6P1.316/PFF0010w* |
| M2830/M2831^a^ | CGATGAACCCTTTGACCCGAC | CCTTTATATCGATCACTAGC | *MAL6P1.316/PFF0010w* |
| 44f/44r new | CCTACACTCACCTCCCCCTA | ACACTCACACGCCTCATCAT | *MAL6P1.4/PFF1580c* |
| M2828/M2829^a^ | GGAGTGGCGTTAGCATTAGG | CACTATCACTATCTCCTTCC | *MAL6P1.4/PFF1580c* |
| 46f/46r | GCGACGCTCAAAAACATTTA | TCATCCAACGCAATCTTTGT | *PF07_0050* |
| M2834/M2835^a^ | ATCCCGAACCCCACGTGAAG | AGGTACATATATATCATTAATATCT | *PF07_0050* |
| 52f/52r | CGTGGTAGTGAAGCACCATC | CCCACCTTCTTGTGGTTTCT | *PF07_0051* |
| M2822/M2823^a^ | ATGTGGAGCGTTGGCATCG | ATCACCACTACTATCTCCTTC | *PF07_0051* |
| 92f/92r | TGCAAGGGTGCTAATGGTAA | CCTGCATTTTGACATTCGTC | *PF08_0103* |
| M2818/M2819^a^ | CTTCGACGATCATGTGGAG | CACTACTATCTCCTTCCATG | *PF08_0103* |
| 7f/7r | GACGGCTACCACAGAGACAA | CGTCATCATCGTCTTCGTTT | *PF11_0008* |
| M2802/M2803 | GAAGTATGTATTGCCCTTGCA | GCGTTCTTGTAGCGCCGACT | *PF11_0008* |
| M2804/M2805 | GTGAAGCCGCCATGTGACAT | GCATATACACACCATCTTCATC | *PF11_0008* |
| M2673/M2674^a^ | CCAGCAGCACCACAACCACC | TTTGCCTTTATATGAACCACTTCTATATGG | *PF11_0008* |
| M2800/M2801^a^ | ACCGCCCTCATGTCTTCTAC | TCAGTACACGCAACAAGTCAACA | *PF11_0008* |
| 10f/10r | TGGTGATGGTACTGCTGGAT | TTTATTTTCGGCAGCATTTG | *PFL0030c* |
| 390/391^a^ | AATAATACCAGTGACATTCTGCAAAA | ACACGTAAAAGGTCCACAGGTG | *PFL0030c* |
