# Supplementary material for: Surface Co-Expression of Two Different PfEMP1 Antigens on Single Plasmodium falciparum-Infected Erythrocytes Facilitates Binding to ICAM1 and PECAM1
Source: PLoS Pathog. 2010 Sep 2;6(9):e1001083. doi: 10.1371/journal.ppat.1001083 (PMC2932717; doi:10.1371/journal.ppat.1001083)
Supplement: Supplementary file 4 [file ppat.1001083.s001.doc]

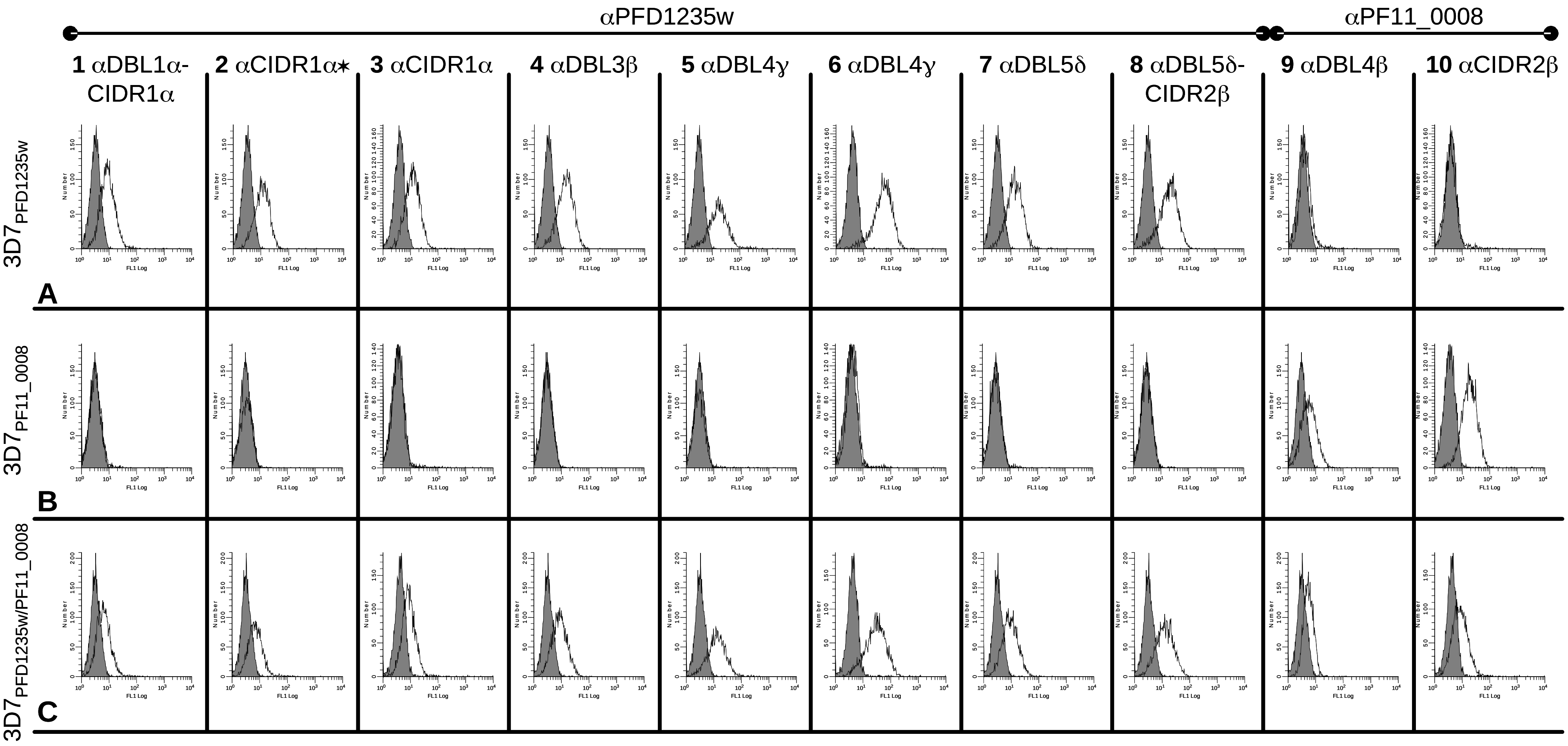
αPFD1235w
αPF11_0008
1 αDBL1α-CIDR1α
2 αCIDR1α✶
3 αCIDR1α
4 αDBL3β
5 αDBL4γ
6 αDBL4γ
7 αDBL5δ
8 αDBL5δ-CIDR2β
9 αDBL4β
10 αCIDR2β
3D7PFD1235w
3D7PF11_0008
3D7PFD1235w/PF11_0008
A
B
C
Number
FL1 Log
