# Supplementary material for: Surface Co-Expression of Two Different PfEMP1 Antigens on Single Plasmodium falciparum-Infected Erythrocytes Facilitates Binding to ICAM1 and PECAM1
Source: PLoS Pathog. 2010 Sep 2;6(9):e1001083. doi: 10.1371/journal.ppat.1001083 (PMC2932717; doi:10.1371/journal.ppat.1001083)
Supplement: Supplementary file 5 [file ppat.1001083.s002.doc]

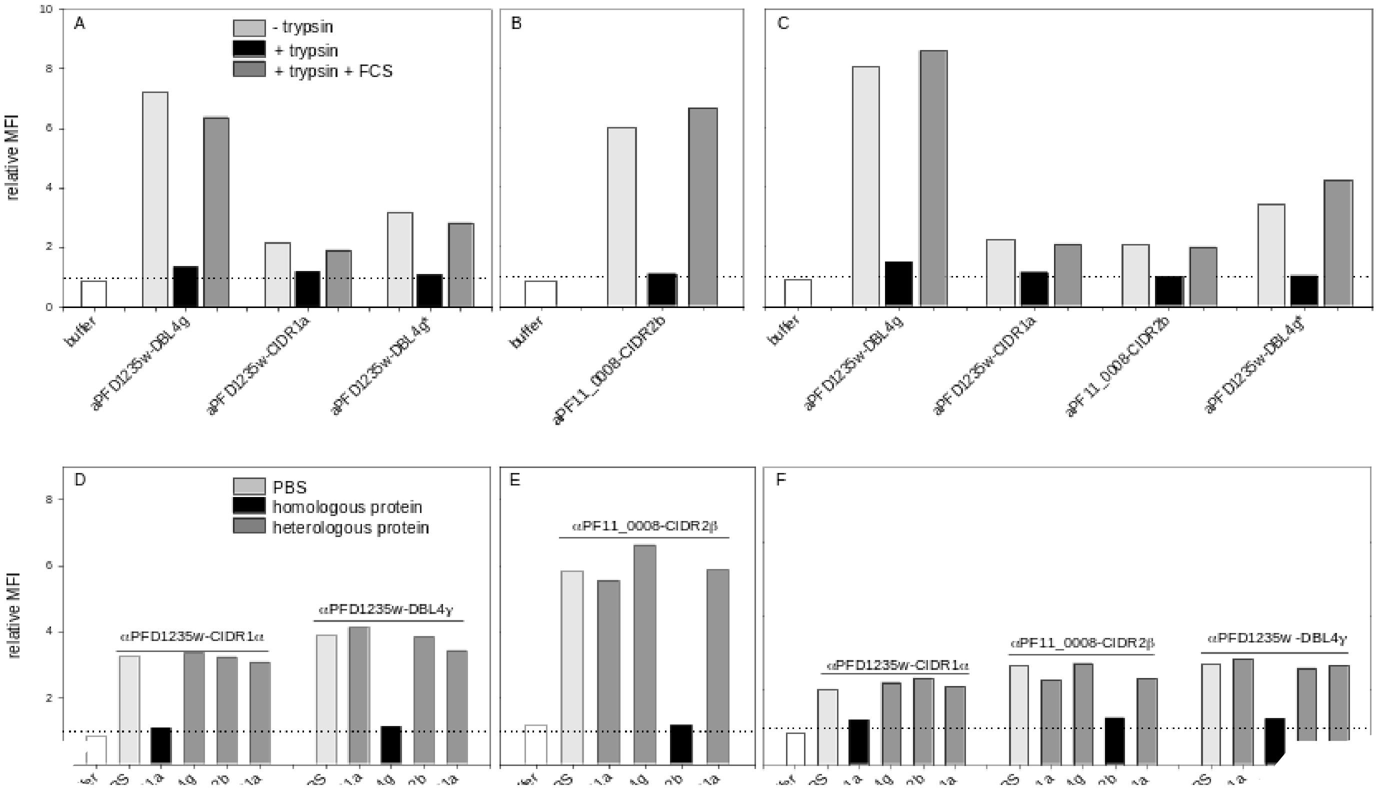
A
B
C
D
E
F
- trypsin
+ trypsin
+ trypsin + FCS
relative MFI
buffer
aPFD1235w-DBL4g
aPFD1235w-CIDR1a
aPFD1235w-DBL4g*
aPF11_0008-CIDR2b
PBS
homologous protein
heterologous protein
αPFD1235w-CIDR1α
αPFD1235w-DBL4γ
αPF11_0008-CIDR2β
αPFD1235w -DBL4γ
